# Et_3_B-mediated and palladium-catalyzed direct allylation of β-dicarbonyl compounds with Morita–Baylis–Hillman alcohols

**DOI:** 10.3762/bjoc.12.234

**Published:** 2016-11-15

**Authors:** Ahlem Abidi, Yosra Oueslati, Farhat Rezgui

**Affiliations:** 1 Université de Tunis EL Manar, Laboratoire de Chimie Organique Structurale et Macromoléculaire, Faculté des Sciences Campus Universitaire, 2092 Tunis, Tunisia

**Keywords:** allylic substitution, Morita–Baylis–Hillman, palladium, triethylborane

## Abstract

A practical and efficient palladium-catalyzed direct allylation of β-dicarbonyl compounds with both cyclic and acyclic Morita–Baylis–Hillman (MBH) alcohols, using Et_3_B as a Lewis acid promoter, is described herein. A wide range of the corresponding functionalized allylated derivatives have been obtained in good yields and with high selectivity.

## Introduction

In nucleophilic allylic substitutions, π-allylpalladium complexes are useful intermediates for the construction of carbon–carbon and carbon–heteroatom bonds in organic synthesis [1]. Usually, palladium species are used as catalysts in the Tsuji–Trost reaction involving, as substrates, allyl carboxylates [2], carbonates [3], and phosphates [4]. Obviously, the direct nucleophilic allylic substitution of allyl alcohols is a more attractive process especially from an economical and environmental point of view [5], as water, generated by this reaction, is a non-toxic byproduct. However, the poor ability of the hydroxy moiety, as a leaving group, has limited the use of the allyl alcohols as substrates. Correlatively, some efforts have been made in this direction by the use of transition metals such as copper [6], nickel [7], ruthenium (I, II) [8], and palladium (0, II) [9–10] as the catalyst or by converting the allylic alcohols into esters of inorganic acids, e.g., As_2_O_3_ [11], B_2_O_3_ [12], CO_2_ [13–14]**.** More recently the Lewis acids, such as, Ti(OiPr )_4_ [15], BEt_3_ [16–19], BPh_3_ [20], SnCl_2_ [21], and FeCl_3_ [22], have also been reported to catalyze these reactions by coordination with the hydroxy moiety, thereby increasing its leaving group ability [23–28].

Recently, Tamaru and co-workers have intensively investigated the use of triethylborane as an additive with either Pd(PPh_3_)_4_ or Pd(OAc)_2_ as catalysts for the allylation of a variety of active methylene compounds [29], aldehydes [30], ketones [31], and imines [32] with only common allylic alcohols.

As part of an ongoing program studying the behavior of MBH derivatives [33] towards β-dicarbonyl compounds, our research group [34–37] has reported an interesting synthesis of bicyclic dienones in a one-pot process involving the reaction of 2-(acetoxymethyl)cyclohex-2-enone with 1,3-dicarbonyl compounds using K_2_CO_3_ as a weak base.

**Figure 1:**
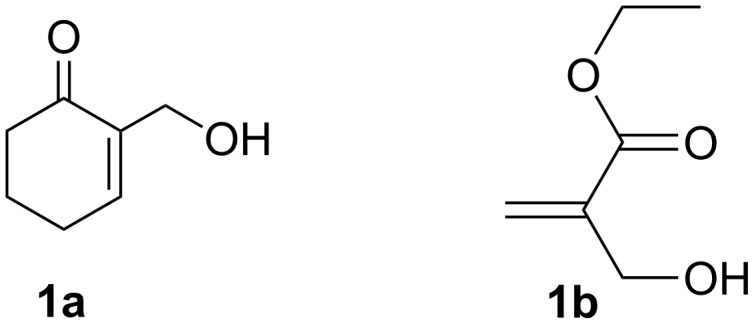
Cyclic and acyclic MBH alcohols.

Later, Chamakh and Amri [38] have described a one-pot synthesis of (*E*)-4-alkylidene-2-cyclohexen-1-ones through a cross coupling of the MBH carboxylates with aliphatic 1,3-diketones in the presence of K_2_CO_3_. A drawback of these synthetic approaches is the need to first perform the acylation step of the corresponding allyl MBH alcohols. For this reason, we herein report an efficient direct method for the allylation of β-dicarbonyl compounds with MBH alcohols [39–40] **1a** and **1b** (Figure 1) considered as multi-functionalized starting materials bearing both allyl alcohol and Michael acceptor moieties.

The interest of this reaction is the fact that it would be possible to directly convert, both cyclic 2-(hydroxymethyl)cyclohex-2-enone (**1a**) and acyclic ethyl 2-(hydroxymethyl)acrylate (**1b**), under the action of 1,3-dicarbonyl compounds **2**, in the presence of an appropriate palladium catalyst and Et_3_B as a Lewis acid promoter, into the allylation compounds **3–8** with the formation of only water as a byproduct. These derivatives can be further used as synthetic intermediates in numerous synthetic routes to heterocyclic compounds and molecules of biological interest [41–42].

## Results and Discussion

We first investigated the allylation of diethyl malonate (**2a**) with the allylic alcohol **1a** in DMF in the presence of Pd(OAc)_2_ (10 mol %), PPh_3_ (20 mol %). Under these conditions, no reaction took place at 80 °C for 3 days (Table 1, entry 1).

**Table 1:** Palladium-catalyzed allylation of diethyl malonate (**2a**) with the MBH alcohol **1a**.

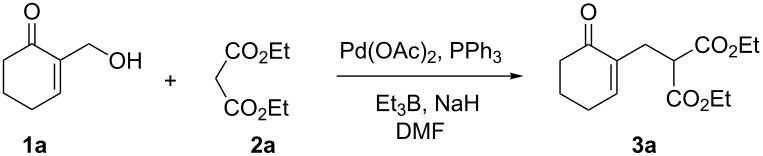				

Entry	NaH (equiv)	Et_3_B (equiv)	*T* (°C), Time (h)	Yield **3a** (%)

1	none	none	80/72	N.R
2	0.5	none	80/24	trace
3	0.5	1	80/12	30
4	1	3	80/6	60

On the other hand, the allylation slowly proceeded in the presence of NaH (0.5 equiv) but only a trace of the expected product **3a** was obtained (Table 1, entry 2). Interestingly, the allylation reaction, carried out in DMF at 80 °C, gave a better result (30% yield in 12 h), using, in addition to NaH (0.5 equiv), 1 equiv of Et_3_B (Table 1, entry 3). Moreover, a remarkable improvement in yield (60% in 6 h) was also observed for the allylation of diethyl malonate (**2a**) with the MBH alcohol **1a** using an excess of Et_3_B (3 equiv) and 1 equiv of NaH (Table 1, entry 4).

### Mechanistic considerations

**Scheme 1:**
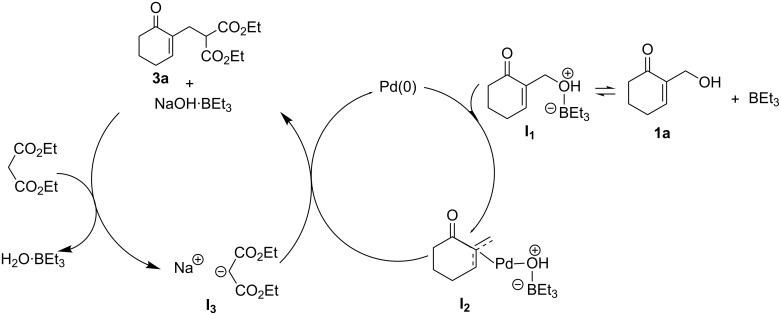
Proposed catalytic cycle involving palladium catalysis for Et_3_B-promoted allylation of diethyl malonate with MBH alcohol **1a**.

Scheme 1 illustrates the most probable catalytic cycle for the allylation of diethyl malonate (**2a**) with allyl alcohol **1a**. We assume that there is first an activation of **1a** through its conversion into **I_1_** using Et_3_B [29], which further gives, in the presence of Pd(0), the π-allylpalladium complex **I_2_**. This intermediate reacts then with the diethyl malonate carbanion **I_3_**, in situ formed, to generate the promoter Et_3_B of this nucleophilic allylic substitution and the desired allylated product **3a**.

Next, under the previously optimized conditions (Table 1, entry 4), we examined the scope of the catalytic system, Et_3_B/ Pd(OAc)_2_/PPh_3_, for a wide range of 1,3-dicarbonyl compounds and related derivatives (p*K*_a_ = 9–14) [29,31] using two typical MBH alcohols **1a** and **1b**. The results of this study are summarized in Table 2.

**Table 2:** Palladium-catalyzed allylation of 1,3-dicarbonyl compounds with MBH alcohol **1a**^a^.

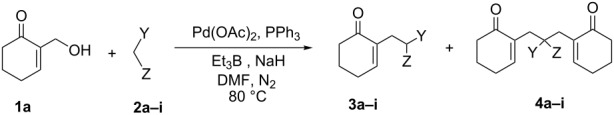				

Entry	Pronucleophile **2**	Time (h)	Yield **3** (%)^a^	Yield **4** (%)^b^

1	CH_2_(CO_2_Et)_2_, **2a**	6	**3a** (60) [48]	–
2	CH_2_(CO_2_Me)_2_, **2b**	6	**3b** (65) [48]	–
3	NCCH_2_CO_2_Et, **2c**	3	**3c** (45) [48]	**4c** (23)
4	MeCOCH_2_CO_2_Me, **2d**	4	**3d** (62) [34,44]	–
5	MeCOCH_2_CO_2_Et, **2e**	6	**3e** (72) [34,44]	–
6	PhCOCH_2_CO_2_Et, **2f**	3	**3f** (76) [34,44]	–
7	MeCOCH_2_COMe, **2g**	3	**3g** (57) [34,44]^c^	
8	PhCOCH_2_COPh, **2h**	3	**3h** (62) [34,44]	
9	PhCOCH_2_COMe, **2i**	3	**3i** (60) [34,44]	

^a^Reaction conditions: dicarbonyl compounds **2** (1.1 mmol), allylic alcohol **1a** (1.0 mmol), Et_3_B (3 mmol), Pd(OAc)_2_ (10 mol %), PPh_3_ (20 mol %), NaH (1 mmol) in DMF (5 mL) under N_2_. ^b^isolated yield; ^c^containing the enolic form **5g**.
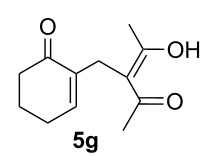

Like diethyl malonate (**2a**), the malonate derivative **2b** (p*K*_a_ = 13) [29] similarly reacted with the allylic alcohol **1a** in the presence of the catalytic system Pd(0)/Et_3_B to exclusively give the mono-allylated product **3a** in 65% yield, whereas the same reaction with ethyl 3-cyano-3-oxopropanoate (**2c**, p*K*_a_ = 10.7) [43] whose acidity is relatively higher, led to a mixture of the mono- and bis-alkylation products **3c** and **4c** in 45 and 23% yields, respectively (Table 2, entries 1–3).

Under the same conditions, the allylation of a variety of β-keto esters and β-diketones (Table 2, entries 4–9), in DMF at 80 °C, selectively gave the monoallylation products **3d–i** in moderate to good yields [44].

The analysis of ^1^H NMR spectra of the β-dicarbonyl derivatives **3a–i** in CDCl_3_ revealed that a keto–enol tautomerism exists only for the acetylacetone derivative **3g** and its enolic form **5g** in a 54:46 ratio, respectively (Table 2, entry 7), whereas the other compounds **3a–f**, **3h** and **3i** are exclusively in the β-dicarbonyl form [45–47].

Moreover, the allylation of ethyl cyclopentanone-2-carboxylate (**2j**) [49] as a cyclic β-keto ester, with alcohol **1a**, catalyzed by the same system, smoothly proceeded at 80 °C in DMF, providing, after 2 h, the mono-allylated product **3j** in 12% yield, along with the tricyclic compound **6j**, in good yield, which is resulting from the intramolecular conjugate addition of **3j** carbanion on the enone moiety (Table 3, entry 2). Under the same conditions, the keto ester **2j** reacted with alcohol **1a,** in DMF at 80 °C for 6 h longer reaction time, to selectively afford the compound **6j** in 76% yield (Table 3, entry 3).

**Table 3:** Palladium-catalyzed allylation of β-keto ester **2j** with the MBH alcohol **1a**^a^.

Entry	Nucleophile	*T* (°C)/Time (h)	Yield (%)

1	**2j**	0 to rt/24	N.R.
2		80/2	**3j**: 12% **6j**: 75%
3		80/6	**6j**: 76%

^a^Reaction conditions: dicarbonyl compounds **2j** (1.1 mmol), allylic MBH alcohol **1a** (1.0 mmol), Et_3_B (3 mmol), Pd(OAc)_2_ (10 mol %), PPh_3_ (20 mol %), NaH (1 mmol) in DMF (5 mL) under N_2_.

**Scheme 2:**
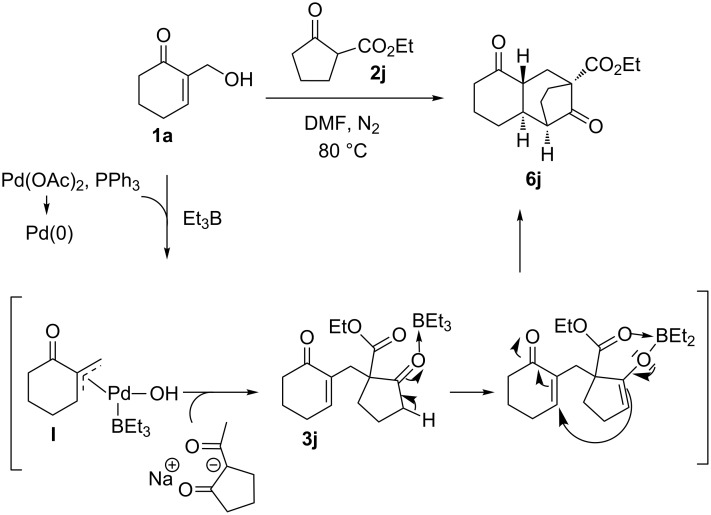
Mechanistic pathway leading to the tricyclic compound **6j**.

A plausible reaction mechanism for the formation of the tricyclic compound **6j** from the MBH alcohol **1a** is presented in Scheme 2. We believe that the treatment of the MBH alcohol **1a** with Et_3_B in the presence of Pd(OAc)_2_ may generate a π-allylpalladium intermediate **I** that further undergoes a nucleophilic substitution reaction with the β-keto ester carbanion derived from **2j**, affording the monoallylated compound **3j**. The conversion of the keto ester **3j** into the tricyclic product **6j** was further performed through an intramolecular conjugate addition of the β-keto ester carbanion onto the enone moiety (Scheme 2) [50–51].

**Figure 2:**
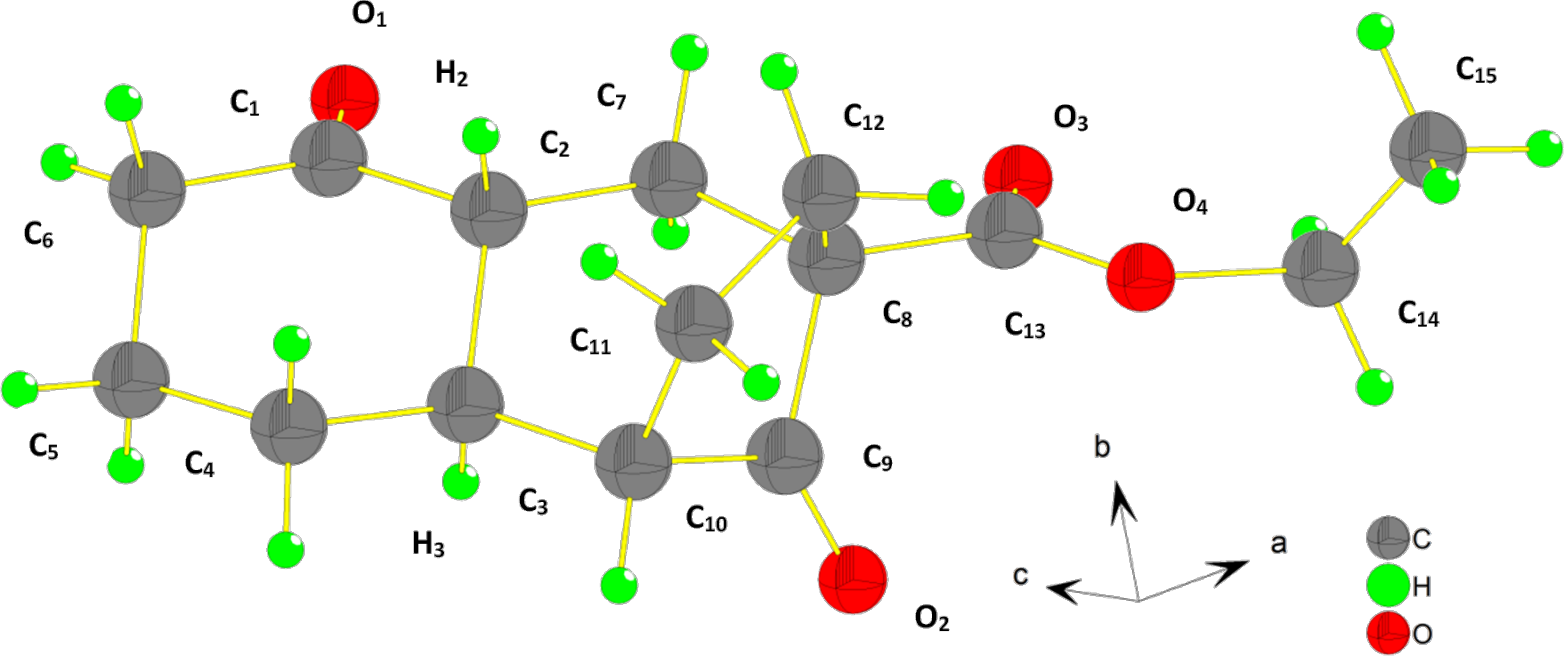
X-ray crystal structure of tricyclic compound **6j**.

The structure of the tricyclic compound **6j** was elucidated on the basis of X-ray diffraction analysis (Figure 2).

In a previous report, Alexakis and co-workers have demonstrated that copper-catalyzed conjugate addition of Grignard reagents onto α-methyl cyclic enones, affording mainly the *trans*-2,3-disubstituted cyclohexanones as being the thermodynamic products [52]. Similarly, we believe that the intramolecular conjugate addition of carbanion **3j** onto α-substituted enone moiety is under thermodynamic control, leading to the tricyclic compound **6j**, in which the two hydrogens H_2_ and H_3_, are on a *trans*-ring junction whereas the second ring junction is *cis* (Figure 2).

Encouraged by these successful results on the allylation of β-dicarbonyl compounds with the alcohol **1a** in the presence of Pd/Et_3_B, we attempted to extend this methodology to the acyclic MBH alcohol **1b** (Table 4). We initially reacted this substrate with ethyl acetoacetate (**2e**, 1.1 equiv) in the presence of the catalyst Pd(OAc)_2_ without any additive. After stirring the reaction mixture for 24 h, either at room temperature or in refluxing THF, the starting alcohol **1b** was recovered (Table 4, entry 1). Next, the use of NaH as a strong base with ethyl acetoacetate (**2e**) allowed the carbanion formation that then reacted with alcohol **1b**, affording a trace of the allylation product **7e** with only a 60% conversion of the starting material (Table 4, entry 2).

**Table 4:** Palladium-catalyzed allylation of ethyl acetoacetate **2e** with the MBH alcohol **1b**.

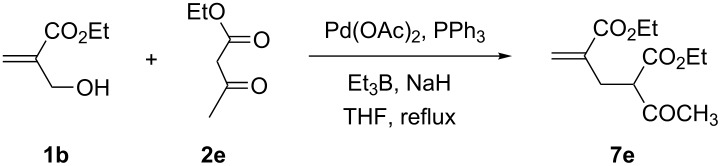				

Entry	NaH (equiv)	Et_3_B (equiv)	*T* (°C)/Time (h)	Conv. **1b** (%)/yield **7e** (%)

1	none	none	rt to reflux/24	N.R.
2	1	none	rt to reflux/24	60/trace
3	1	1	reflux	100/30
4	1	2	rt/20	100/60
5	1	2	reflux/2	100/60

The addition of a Lewis acid (1 equiv of Et_3_B) started the reaction by activating the hydroxy group, affording the allylation product **7e** in 30% isolated yield (Table 4, entry 3). To further improve the performance of this allylation reaction, we employed 2 equiv of Et_3_B (Table 4, entry 4). The reaction afforded within 20 h, at room temperature, the compound **7e** in 60% yield. Interestingly, in refluxing THF, this reaction was achieved in shorter reaction time (2 h), leading to the same compound in the same yield (Table 4, entry 5).

Encouraged by these results and those of cyclic MBH alcohol **1a**, we selected the acyclic alcohol **1b** to react with stabilized carbanions derived from the β-keto esters **2e–f** and β-diketones **2g–i** in anhydrous THF using 1 equiv of NaH and 2 equiv of Et_3_B. Under these conditions, all these reactions worked well in refluxing THF, affording in 2 h the corresponding monoallylation products **7e–i** [41,44] in 60–70% yields (Table 5, entries 1–4).

**Table 5:** Palladium-catalyzed allylation of a variety of β-dicarbonyl compounds with the MBH alcohol **1b**.

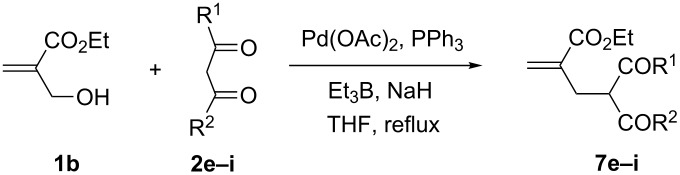				

Entry	β-Dicarbonyl compound **2**	Time (h)	Compound **7**	Yield **7** (%)

1	MeCOCH_2_CO_2_Et, **2e**	2	**7e** [41,44]	60
2	Ph COCH_2_CO_2_Et, **2f**	2	**7f** [41,44]	68
3	MeCOCH_2_COMe, **2g**	2	**7g** [41,44]	65^a^
4	Ph COCH_2_COMe, **2i**	2	**7i** [41,44]	70

^a^Containing 30% of the enolic form **8g**.
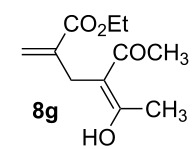

## Conclusion

In summary, we have developed a mild and direct process for the C–C bond formation from the reaction of the MBH alcohols **1a** and **1b** with β-dicarbonyl compounds **2** in the presence of a palladium catalyst and Et_3_B (promoter) with the formation of only water as a side product. This method provides a straightforward and practical route to a range of allylated compounds. Further work is in progress in our laboratory to investigate the Pd catalysis of the reaction of the MBH adducts with various pronucleophiles including nitroalkanes, amines and thiols.

## Experimental

### General

IR spectra were recorded on a Bruker IFS 66v/S spectrometer. ^1^H NMR and ^13^C NMR spectra were recorded either on a Bruker AC-300 spectrometer (300 MHz for ^1^H and 75 MHz for ^13^C) in CDCl_3_, using TMS as an internal standard (chemical shifts in δ values, *J* in Hz). Mass spectra (EI) were recorded on an Hewlett-Packard (70 eV) apparatus. Analytical thin-layer chromatography (TLC) was performed using Fluka Kieselgel 60 F254 precoated silica gel plates. Visualization was achieved by UV light (254 nm). Flash chromatography was performed using Merck silica gel 60 and a gradient solvent system (petroleum ether/ether as eluents).

### General procedure for the allylation of β-dicarbonyl compounds with the MBH alcohols **1a** and **1b**

Into a nitrogen-purged two-necked flask, equipped with a reflux condenser, containing 5 mL of DMF or THF, Pd(OAc)_2_ (10 mol %), and PPh_3_ (20 mol %) were added successively the 1,3-dicarbonyl compound **2** (1.1 mmol), NaH (1 mmol) and an Et_3_B solution 1.0 M (2 to 3 mmol) in THF. The mixture was stirred for 5 to 10 min at room temperature, then the MBH allylic alcohol **1a** or **1b** (1 mmol) was added. The mixture was stirred and heated at 80 °C for 3 to 6 h during which the mixture was colored black. When the reaction, followed by TLC, was finished, the mixture was diluted with CH_2_Cl_2_ (20 mL) and washed with 2 M HCl, sat. NaHCO_3_ and then brine. The organic extracts were dried over MgSO_4_ and concentrated in vacuo and the residual oil was subjected to column chromatography over silica gel (gradient: petroleum ether/ether = 4:1) to give the pure allylated products **3–8** in moderate to good yields.

## Supporting Information

File 1 Experimental procedures, characterization and spectral data for synthesized compounds and X-ray data for compound **6j**.
