# Supplementary material for: Et3B-mediated and palladium-catalyzed direct allylation of β-dicarbonyl compounds with Morita–Baylis–Hillman alcohols
Source: Beilstein J Org Chem. 2016 Nov 15;12:2402–9. doi: 10.3762/bjoc.12.234 (PMC5238553; doi:10.3762/bjoc.12.234)
Supplement: File 1 — Experimental procedures, characterization and spectral data for synthesized compounds and X-ray data for compound 6j. [file Beilstein_J_Org_Chem-12-2402-s001.pdf]

Supporting Information

for

Et_3B-mediated and palladium-catalyzed direct allylation of β-dicarbonyl compounds with Morita–Baylis–Hillman alcohols

Ahlem Abidi, Yosra Oueslati and Farhat Rezgui*

Address: Université de Tunis EL Manar, Laboratoire de Chimie Organique Structurale et Macromoléculaire, Faculté des Sciences Campus Universitaire, 2092 Tunis, Tunisia

Email: Farhat Rezgui - rez_far@yahoo.fr

*Corresponding author

Experimental procedures, characterization and spectral data for synthesized compounds and X-ray data for compound 6j

Table of Contents:

Analytical data, ^1H NMR and ^{13}C NMR spectra …………………………...Page S2–S29.

GC–MS data ……………………..……………………………………………...Page S30–S37.

Crystallographic analysis of 6j and references …………………………….……Page S38.

2-(6-Oxo-cyclohex-1-enylmethyl)malonic acid diethyl ester (3a) [1]

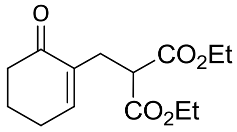

3a

Yield: 60%; yellow oil; ^{1}H NMR (300 MHz, $CDCl_3$): 6.83 (t, *J*=4.5 Hz, 1H), 4.17 (q, *J*=7.5 Hz, 4H), 3.65 (t, *J*=8.0 Hz, 1H), 2.75 (d, *J*= 8.0 Hz, 2H), 2.44–2.40 (m, 2H), 2.38–2.33 (m, 2H), 2.02–1.94 (m, 2H), 1.25 (t, *J*=7.5 Hz, 6H); ^{13}C NMR (75 MHz, $CDCl_3$): 198.8, 169, 148.4, 135.8, 61.3, 50.7, 38.3, 29.8, 26.1, 22.9, 14.1; MS (m/z): 55 (34), 109 (25), 148 (75), 176 (100), 194 (42), 222 (78), 268 (M^+, 4).

2-(6-Oxo-cyclohex-1-enylmethyl)malonic acid dimethyl ester (3b) [1]

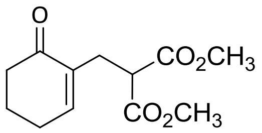

3b

Yield: 65%; yellow oil; ^{1}H NMR (300 MHz, $CDCl_3$): 6.84 (t, *J*=4.5 Hz, 1H), 3.78–3.63 (m, 7H), 2.75 (d, *J*=9.0, 2H), 2.44–2.39 (m, 2H), 2.38–2.33 (m, 2H), 2.02–1.93 (m, 2H); ^{13}C NMR (75 MHz, $CDCl_3$): 198.5, 169.4, 148.4, 135.6, 52.3, 50.5, 37.9, 30.0, 26.2, 22.9; MS (m/z): 109 (37), 121 (89), 148 (74), 176 (97), 208 (100), 222 (3), 240 (M^+, 4).

Ethyl 2-cyano-3-(6-oxocyclohex-1-en-1-yl)propanoate (3c) [1]

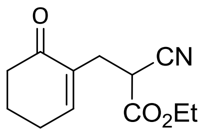

3c

Yield: 45% ; yellow oil; ^{1}H NMR (300 MHz, $CDCl_3$): 7.01 (t, *J*=4.0 Hz, 1H), 4.25 (q, *J*=7.5 Hz, 2H), 3.90 (t, *J*=9 Hz, 1H), 2.98–2.54 (m, 2H), 2.48–2.44 (m, 4H), 2.07–2.03 (m, 2H), 1.32 (t, *J*=7.5 Hz, 3H); ^{13}C NMR (75 MHz, $CDCl_3$): 198.8, 165.7, 150.5, 134.0, 116.3, 62.8, 38.1, 36.8, 31.1, 26.2, 22.8, 14.0; MS (m/z): 81 (85), 109 (24), 147 (100), 175 (60), 192 (11), 221 (M^+, 64).

Ethyl2-cyano-3-(6-oxocyclohex-1-en-1-yl)-2-(6-oxocyclohex-1-en-1yl)methyl)propanoate (4c)

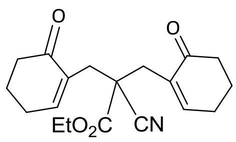

4c

Yield: 23% ; yellow oil; ^{1}H NMR (300 MHz, $CDCl_3$): 7.03 (t, *J*=4.5 Hz, 2H), 4.18 (q, *J*=7.5 Hz, 2H), 2.81 (AB, *J*=15.0 Hz, 4H), 2.48–2.41 (m, 8H), 2.06–1.97 (m, 4H), 1.30 (t, *J*=7.5 Hz, 3H); ^{13}C NMR (75 MHz, $CDCl_3$): 198.1, 167.9, 150.2, 133.9, 118.4, 62.8, 50.4, 38.0, 35.2, 26.3, 22.7, 14.0; MS (m/z): 53 (83), 110 (47), 174 (100), 220 (41), 256 (50), 329 (M^+, 18).

3-Oxo-2-(6-oxo-cyclohex-1-enylmethyl)-butyric acid methyl ester (3d) [2,3]

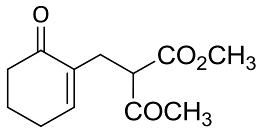

3d

Yield : 62%; yellow oil; IR ($CHCl_3$): 1742, 1714, 1667 cm^{-1}; ^{1}H NMR (300 MHz, $CDCl_3$): 6.83 (t, *J*=4.5 Hz, 1H), 3.81–3.74 (m, 1H), 3.70 (s, 3H), 2.80–2.59 (m, 2H), 2.43–2.39 (m, 2H), 2.37–2.31 (m, 2H), 2.22 (s, 3H), 2.01–1.98 (m, 2H); ^{13}C NMR (75 MHz, $CDCl_3$): 202.5, 199.1, 169.8, 148.6, 136.0, 58.1, 52.3, 38.4, 29.3, 29.1, 26.2, 22.9; MS (m/z): 55 (30), 66 (19), 94 (36), 109 (13), 122 (100), 149 (92), 206 (53), 224 (M^+, 0.5). Anal. Calcd for $C_{12}H_{16}O_4$: C, 64.29, H, 7.14. Found: C, 64.23, H, 7.16.

3-Oxo-2-(6-oxo-cyclohex-1-enylmethyl)-butyric acid ethyl ester (3e) [2,3]

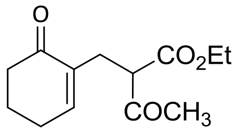

3e

Yield : 72%; yellow oil; IR($CHCl_3$): 1740, 1710, 1660 cm^{-1}; ^{1}H NMR (300 MHz, $CDCl_3$): 6.84 (t, *J*=3.0 *Hz*, 1H), 4.17 (q, *J*=7.5 Hz, 2H), 3.80–3.75 (m, 1H), 2.80–2.57 (m, 2H), 2.44–2.41 (m, 2H), 2.39–2.33 (m, 2H), 2.23 (s, 3H), 2.01–1.93 (m, 2H), 1.25 (t, *J*=7.5 Hz, 3H); ^{13}C NMR (75 MHz, $CDCl_3$): 202.5, 199.0, 169.2, 148.5, 135.8, 61.1, 58.1, 38.2, 29.2, 28.9, 26.0, 22.8, 14.0; MS (m/z): 55 (32), 66 (52), 77 (43), 79 (40), 91 (39), 94 (58), 107 (7), 109 (19), 122 (100), 123 (36), 136 (10), 149 (74), 150 (32), 192 (8), 193 (20), 220 (64).

3-Oxo-2-(6-oxo-cyclohex-1-enylmethyl)-3-phenyl-propionic acid ethyl ester (3f) [3]

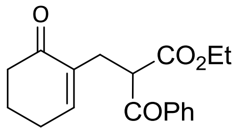

3f

Yield : 76%; yellow oil; IR ($CHCl_3$): 1737, 1699, 1600, 1448 cm^{-1}; ^{1}H NMR (300 MHz, $CDCl_3$): 8.06–8.03 (m, 2H), 7.6–7.55 (m, 1H), 7.49–7.44 (m, 2H), 6.88 (t, *J*=4.5 Hz, 1H), 4.74–4.69 (m, 1H), 4.13 (q, *J*=7.5 *Hz*, 2H), 2.85–2.81 (m, 2H), 2.40–2.33 (m, 2H), 2.30–2.26 (m, 2H), 1.97–1.79 (m, 2H), 1.17 (t, *J*=7.5 *Hz*, 3H); ^{13}C NMR (75 MHz, $CDCl_3$): 199.5, 195.6, 169.5, 149.4, 136.3, 135.9, 133.5, 128.8, 128.7, 61.2, 52.6, 38.4, 30.7, 26.1, 22.9, 14.1; MS (m/z): 77 (33), 105 (100), 121 (5), 149 (22), 195 (14), 226 (5), 254 (22), 255 (15), 282 (45), 300 (M^+, 2).

3-(6-Oxo-cyclohex-1-enylmethyl)-pentane-2,4-dione (3g) and its enolic form (5g) [2,3]

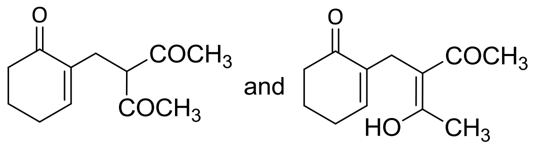

3g and **5g**

Yield : 57 %; yellow oil; IR ($CHCl_3$): 3500, 1725, 1700, 1670 cm^{-1}; ^{1}H NMR (keto, $CDCl_3$): 6.82 (t, *J*=4.5 Hz, 1H), 3.96 (t, *J*=8.0 Hz, 1H), 2.68 (d, *J*=8.0 Hz 2H), 2.50–2.46 (m, 2H), 2.38–2.31 (m, 2H), 2.18 (s, 6H), 1.98–1.93 (m, 2H); ^{1}H NMR (enol, $CDCl_3$): 6.56–6.53 (m,

1H), 3.17 (q, J=3.0 Hz, 2H), 2.43–2.39 (m, 2H), 2.36–2.33 (m, 2H), 2.02 (s, 6H), 1.98–1.93 (m, 2H); ^{13}C NMR (keto, $CDCl_3$): 203.9, 199.4, 148.7, 136.0, 66.5, 38.4, 29.6, 26.6, 26.1, 26.0; ^{13}C NMR (enol, $CDCl_3$): 199.4, 199.2, 191.9, 144.6, 137.0, 106.1, 38.4, 28.8, 26.1, 26.0, 23.0, 22.9. Anal. Calcd for $C_{12}H_{16}O_4$: C, 69.30, H = 7.69. Found: C = 69.37, H = 7.73.

2-(6-Oxo-cyclohex-1-enylmethyl)-1,3-diphenyl-propane-1,3-dione (3h) [2,3]

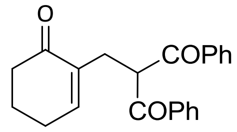

3h

Yield: 62%; white solid; m.p. 118-120°C; IR ($CHCl_3$): 1690, 1660, 1600, 1440 cm^{-1}; ^{1}H NMR (300 MHz, $CDCl_3$): 8.07–8.04 (m, 4H), 7.58–7.42 (m, 6H), 7.00 (t, J=4.5 Hz, 1H), 5.75 (t, J=7.0 Hz, 1H), 2.96 (d, J=7.0 Hz, 2H), 2.38–2.33 (m, 2H), 2.25–2.20 (m, 2H), 1.84–1.76 (m, 2H); ^{13}C NMR (75 MHz, $CDCl_3$): 200.3, 196.1, 150.9, 136.1, 135.8, 133.5, 128.9, 128.9, 54.2, 38.4, 31.3, 26.1, 22.7; MS (m/z): 51 (24), 77 (100), 105 (100), 210 (61), 227 (81), 314 (32), 332 (M^+, 1). Anal. Calcd. For $C_{22}H_{20}O_3$: C, 79.52 ; H, 6.02. Found : C, 79.50 ; H, 6.00.

2-(6-Oxo-cyclohex-1-enylmethyl)-1-phenyl-butane-1,3-dione (3i) [2,3]

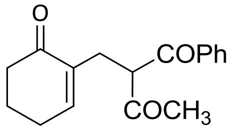

3i

Yield: 60%; yellow oil; IR ($CHCl_3$): 1720, 1680, 1670 cm^{-1}; ^{1}H NMR (300 MHz, $CDCl_3$): 8.04–8.01 (m, 2H), 7.62–7.59 (m, 1H), 7.51–7.46 (m, 2H), 6.85 (t, J=4.5 Hz, 1H), 4.87 (t, J=6.0 Hz, 1H), 2.90–2.72 (m, 2H), 2.38–2.34 (m, 4H), 2.33–2.25 (m, 2H), 2.14 (s, 3H), 1.91–1.81 (m, 2H); ^{13}C NMR (75 MHz, $CDCl_3$): 203.3, 199.7, 196.9, 149.5, 136.5, 135.8, 133.7, 128.8, 128.5, 60.4, 38.4, 30.3, 29.2, 26.1, 22.8; MS (m/z): 51 (8), 55 (7), 77 (51), 105 (100), 106 (13), 123 (24), 148 (12), 165 (15), 210 (12), 211 (6), 227 (21), 252 (18), 270 (M^+, 0.5).

Ethyl 2-oxo-1-(6-oxocyclohex-1-en-1-yl)methyl)cyclopentanecarboxylate (3j)

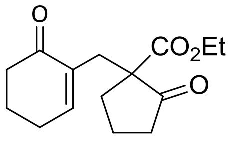

3j

Yield: 12%; colorless crystal; m.p. 80-82°C; ^{1}H NMR (300 MHz, $CDCl_3$): 6.75 (t, J=4.5 Hz, 1H), 4.15 (q, J=7.5 Hz, 2H), 2.93 (AB, J= 12 Hz, 2H), 2.35–2.2 (m, 6H), 2.01–1.88 (m, 6H), 1.27 (t, J=7.5 Hz, 3H); ^{13}C NMR (75 MHz, $CDCl_3$): 213, 199.3, 170.9, 149.1, 135.3, 61.2, 60.7, 38.1, 37.9, 31.2, 28.5, 25.8, 22.7, 20.5, 14.1; MS (m/z): 55 (49), 109 (25), 163 (100), 190 (52), 218 (23), 236 (63), 264 (M^+, 9).

1,10-Dioxo-decahydro-5,8-methano-benzocycloheptene-8-carboxylic acid ethyl ester (6j)

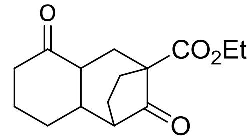

6j

Yield: 76%; colorless crystal; IR ($CHCl_3$): 2953, 1759, 1725, 1460 cm^{-1}; m.p. 116-120°C; ^1H NMR (300 MHz, $CDCl_3$): 4.17 (q, J=9.0 Hz, 2H), 2.72–2.56 (m, 2H), 2.4–2.35 (m, 4H), 2.17–2.11 (m, 3H), 2.08–2.01 (m, 2H), 1.95–1.67 (m, 4H), 1.28 (t, J=9.0 Hz, 3H); ^{13}C NMR (75 MHz, $CDCl_3$): 212.3, 209.5, 170.6, 61.2, 56.8, 51.2, 50.3, 47.3, 41.2, 36.7, 29.3, 27.6, 25.9, 17.6, 14.2; MS (m/z): 55 (67), 123 (100), 162 (89), 190 (72), 236 (73), 264 (M^+, 56).

Diethyl 2-acetyl-4-methylenepentanedioate (7e) [3,4]

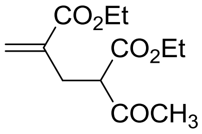

7e

Yield: 60%; yellow oil; IR($CHCl_3$): 1740, 1710, 1660 cm^{-1}; ^1H NMR (300 MHz, $CDCl_3$): 6.21 (s, 1H), 5.64 (s, 1H), 4.26–4.15 (m, 4H), 3.85–3.80 (m, 1H), 2.91–2.76 (m, 2H), 2.25 (s, 3H), 1.33–1.24 (m, 6H) ^{13}C NMR (75 MHz, $CDCl_3$): 202.1, 169.0, 166.4, 137.0, 127.7, 61.4, 60.9, 58.4, 30.6, 29.2, 14.2, 14.1; MS (m/z): 53 (18), 98 (100), 126 (78), 151 (48), 200 (M-43, 43).

Diethyl 2-benzoyl-4-methylenepentanedioate (7f) [3,4]

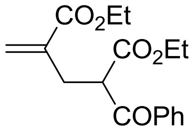

7f

Yield: 68%; yellow oil; IR ($CHCl_3$): 1737, 1699, 1600, 1448 cm^{-1}; ^1H NMR (300 MHz, $CDCl_3$): 8.05–8.03 (m, 2H), 7.60–7.44 (m, 3H), 6.21 (s, 1H), 5.71 (s, 1H), 4.75 (t, J=8.0 Hz, 1H), 4.22 (q, J=7.5Hz, 2H), 4.13 (q, J=6.0 Hz, 2H), 2.99 (d, J=8.0 Hz, 2H), 1.30 (t, J=7.5 Hz, 3H), 1.16 (t, J=6.0 Hz, 3H) ; ^{13}C NMR (75 MHz, $CDCl_3$): 194.7, 169.2, 166.5, 136.8, 136.1, 133.6, 128.7, 128.3, 128.2, 61.4, 60.9, 53.0, 31.9, 14.2, 14.0; MS (m/z): 51 (4), 77 (22), 105 (100), 304 (M^+, 0.4).

Ethyl 4-acetyl-2-methylene-5-oxohexanoate (7g) and its enolic form (8g) [3,4]

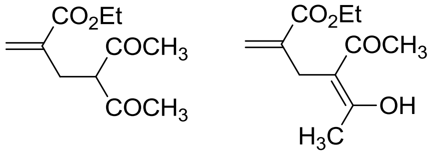

7g and **8g**

Yield: 65%; red oil; IR ($CHCl_3$): 1710, 1630, 1607 cm^{-1}; ^1H NMR (keto, $CDCl_3$): 6.20 (s, 1H), 5.63 (s, 1H), 4.29–4.19 (m, 2H), 4.01 (t, J=6.0 Hz, 1H), 2.83 (d, J=6.0 Hz, 2H), 2.20 (s, 6H), 1.36 (m, 3H); ^1H NMR (enol, $CDCl_3$): 6.25 (s, 1H), 5.44 (s, 1H), 4.29–4.19 (m, 2H), 3.27 (s, 2H), 2.06 (s, 6H), 1.36 (m, 3H); ^{13}C NMR (keto, $CDCl_3$): 203.2, 166.3, 137.1, 127.6, 66.7, 60.9, 31.9, 29.7, 14.2; ^{13}C NMR (enol, $CDCl_3$): 203.2, 192.0, 166.8, 138.8, 124.2, 106.1, 60.9, 29.5, 22.8, 14.2; MS (m/z): 53 (21), 95 (79), 123(100), 141 (43), 169 (59), 212 (M^+, 15).

Ethyl 4-benzoyl-2-methylene-5-oxohexanoate (7i) [3,4]

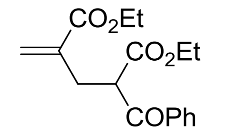

7i

Yield: 70%; yellow oil; IR ($CHCl_3$): 1713, 1676, 1630, 1446 cm^{-1}; ^1H NMR (300 MHz, $CDCl_3$): 8.03–8.00 (m, 2H), 7.62–7.39 (m, 3H), 6.18 (s, 1H), 5.67 (s, 1H), 4.91 (t, *J*=8.0 Hz, 1H), 4.21 (q, *J*=7.5 Hz, 2H), 2.98 (d, *J*=8.0 Hz, 2H), 2.15 (s, 3H), 1.29 (t, *J*=7.5Hz, 3H); ^{13}C NMR (75 MHz, $CDCl_3$): 202.9, 195.9, 166.5, 136.8, 136.3, 133.8, 128.8, 128.3, 127.1, 61.0, 60.9, 31.5, 28.8, 14.1; MS (m/z): 51 (5), 77 (26), 105 (100), 158 (6), 274 (M^+, 0.4).

3a ^1H NMR (300 MHz, $CDCl_3$)

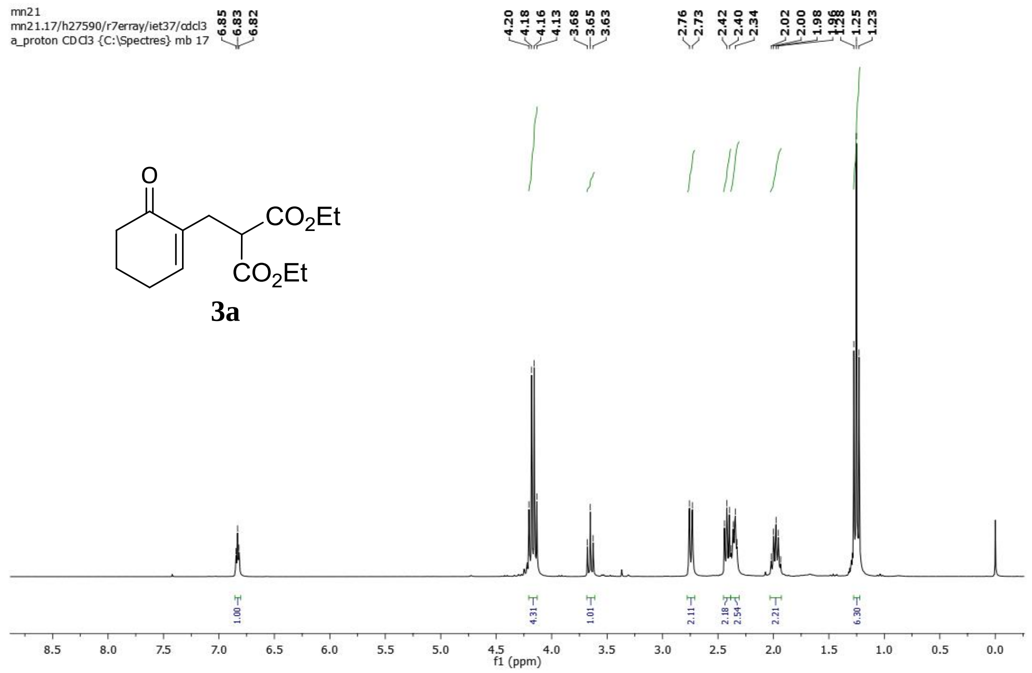

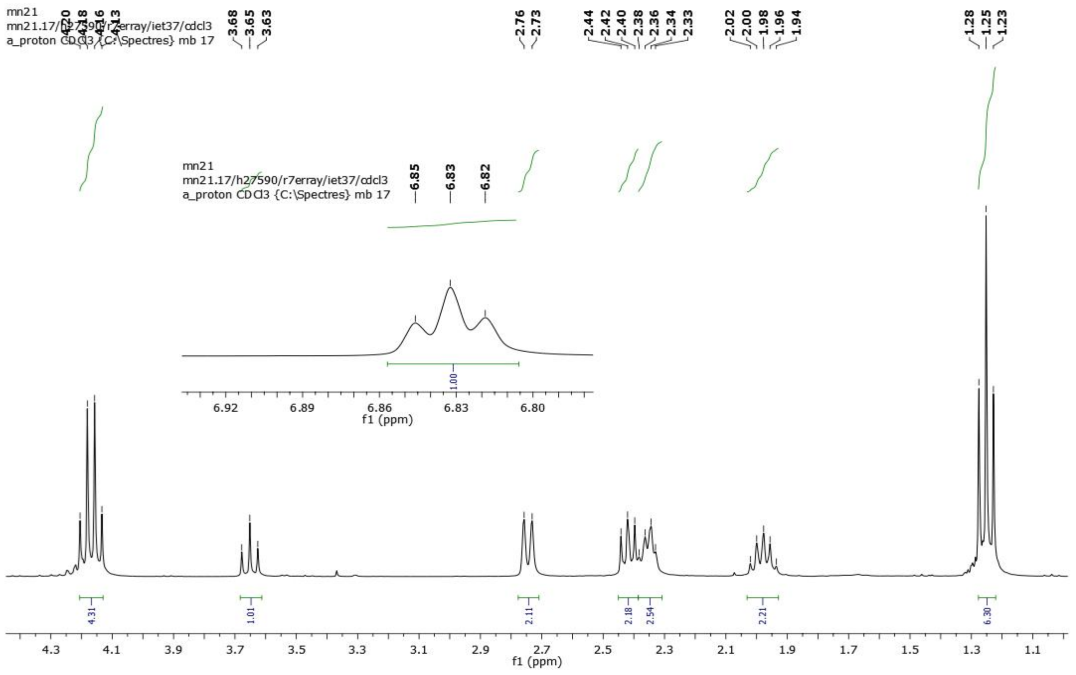

^{13}C NMR (75 MHz, $CDCl_3$)

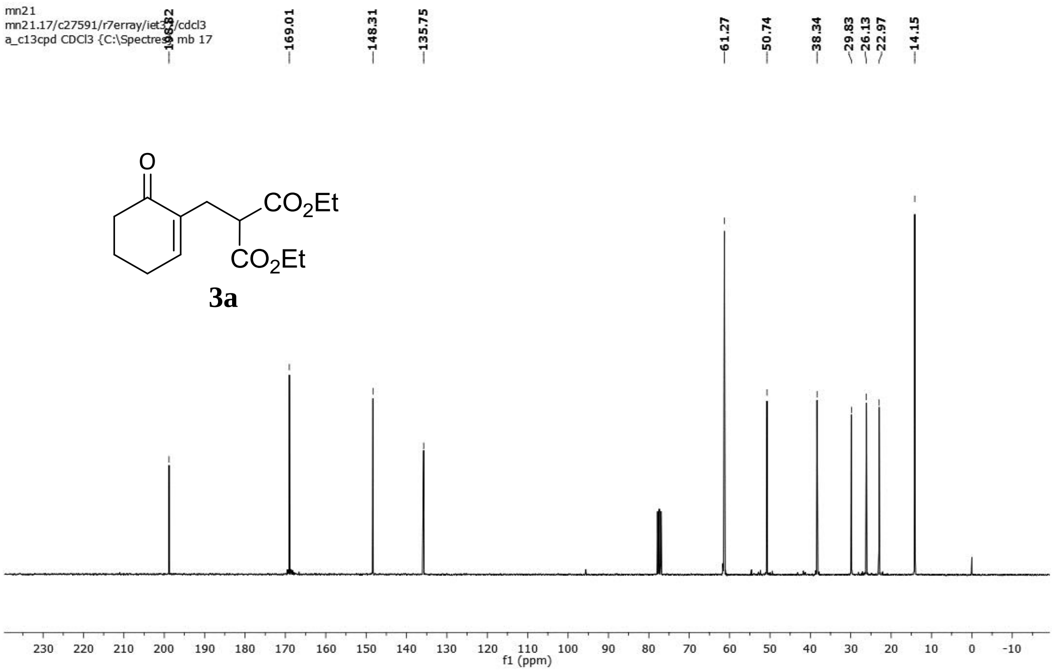

3b ^{1}H NMR (300 MHz, $CDCl_3$)

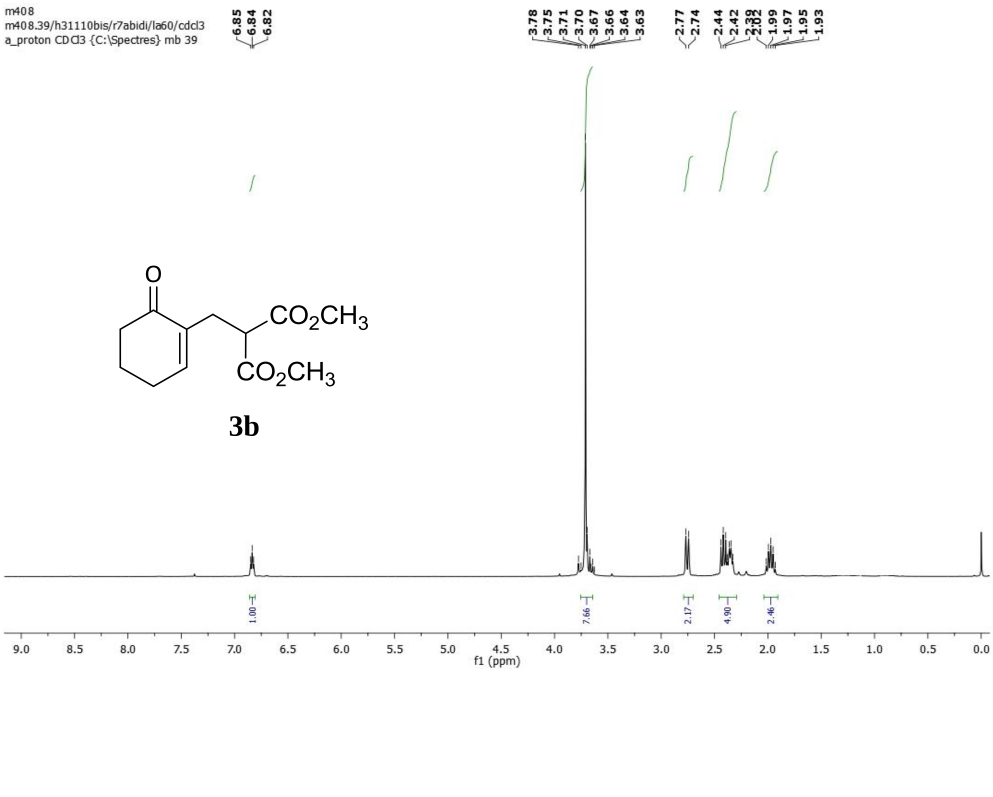

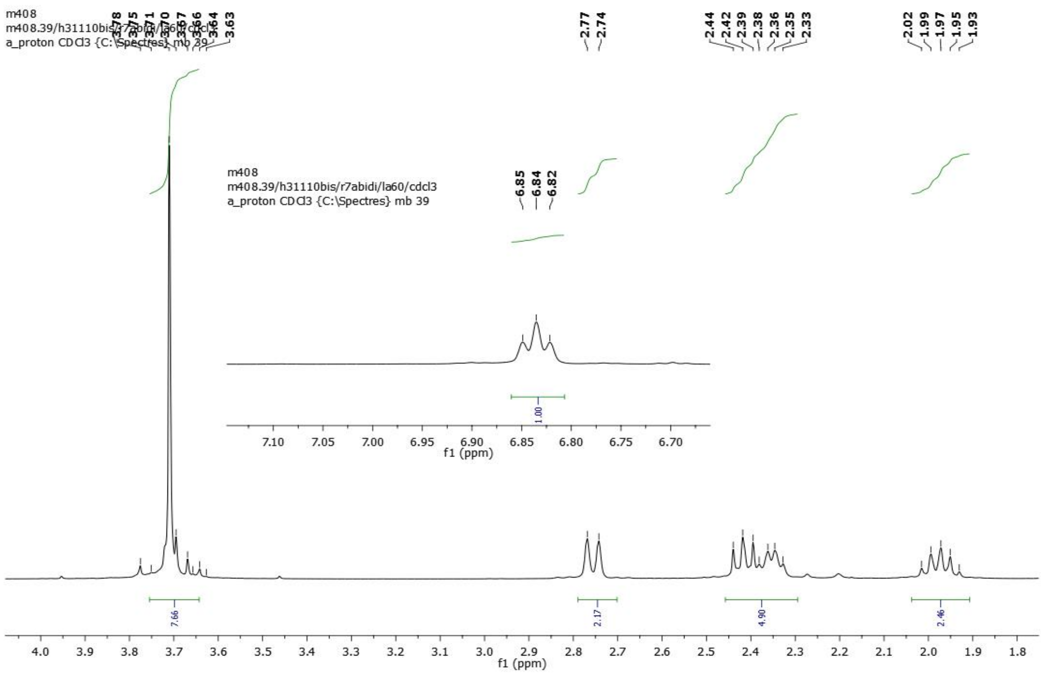

^{13}C NMR (75 MHz, $CDCl_3$)

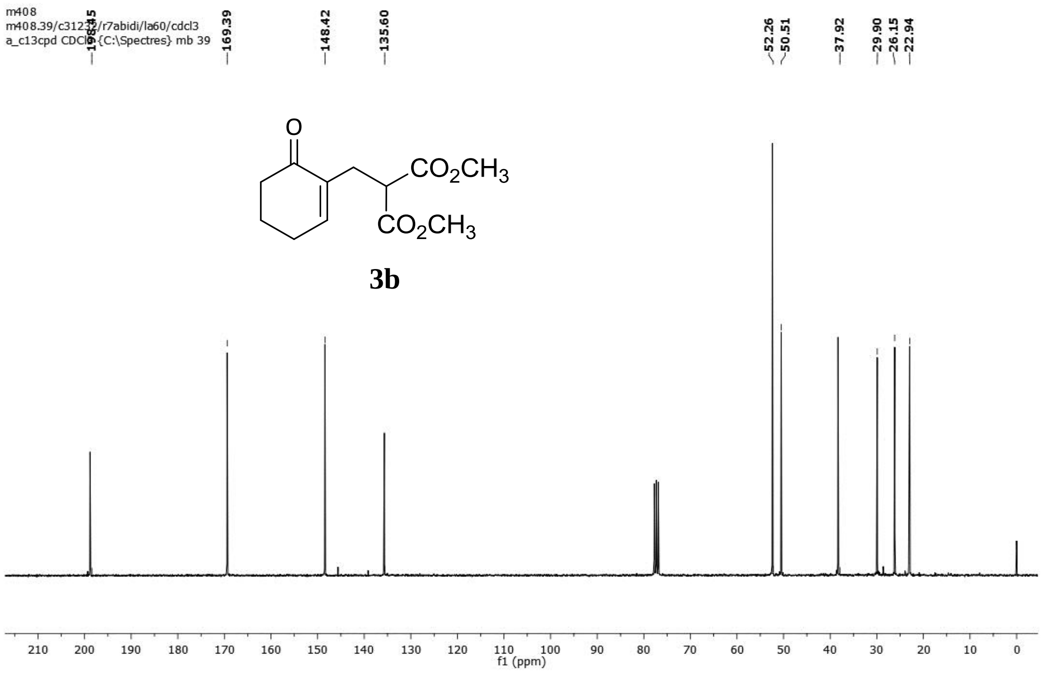

3c ^{1}H NMR (300 MHz, $CDCl_3$)

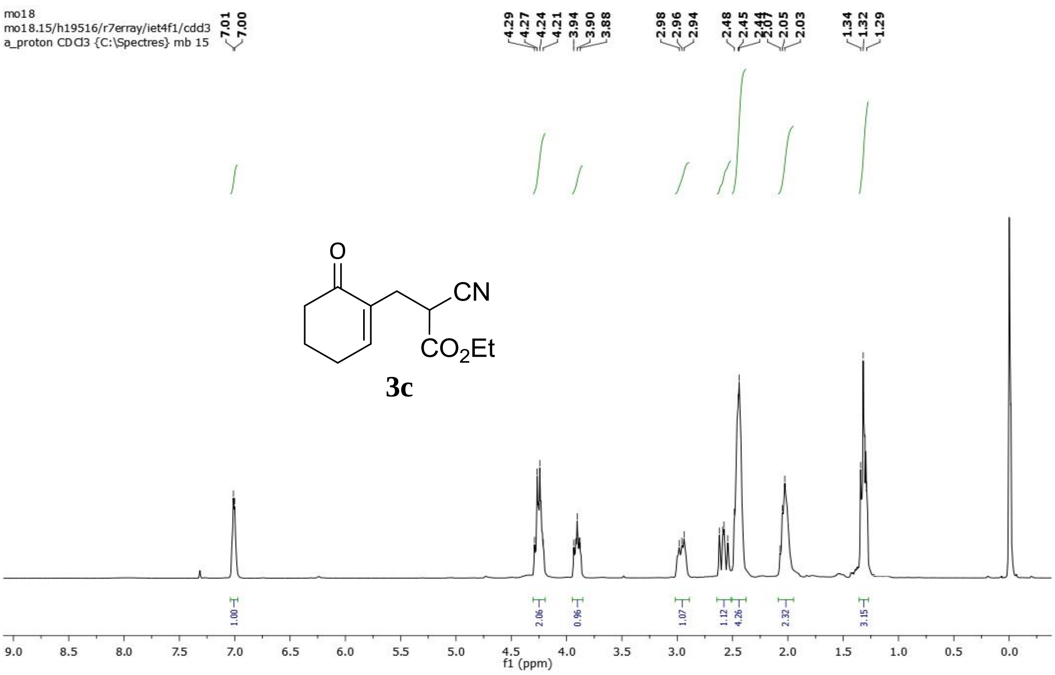

^{13}C NMR (75 MHz, $CDCl_3$)

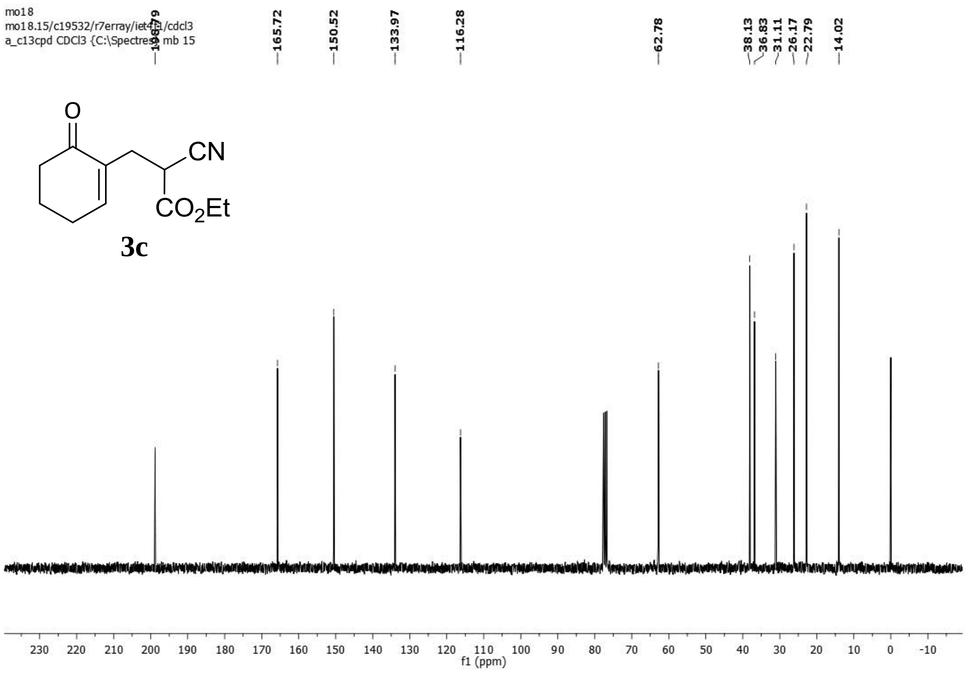

4c ^{1}H NMR (300 MHz, $CDCl_3$)

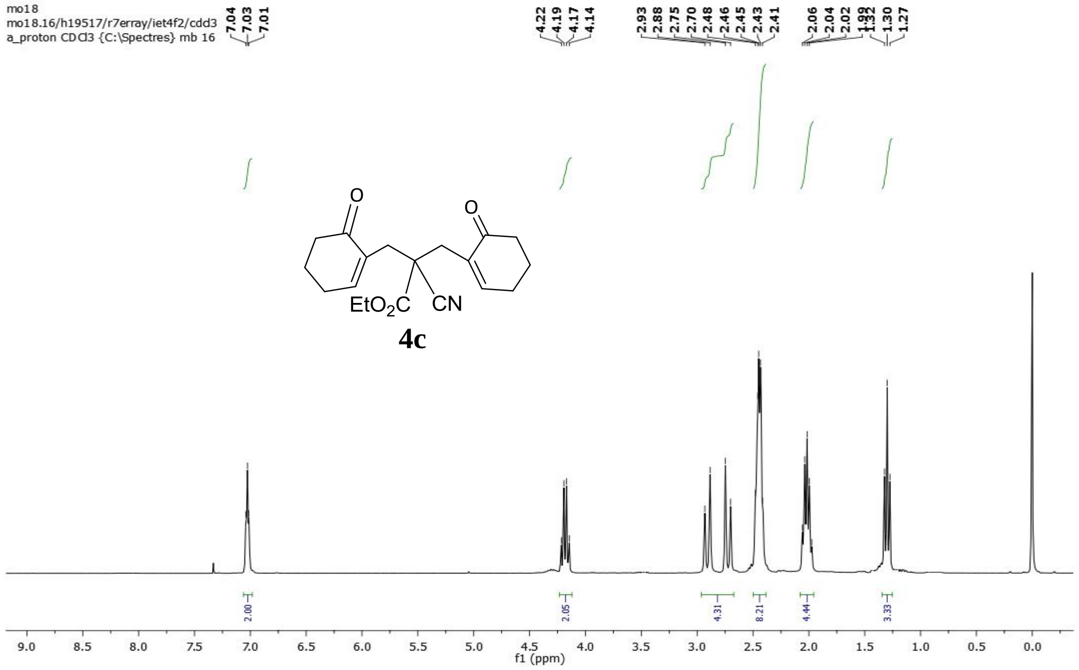

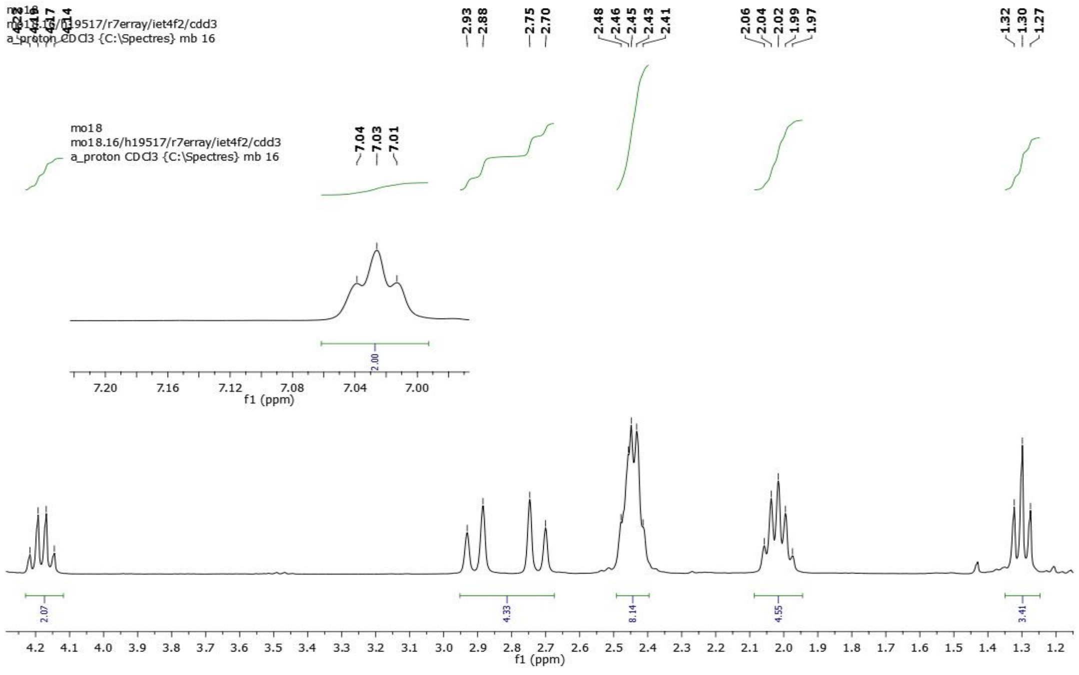

^{13}C NMR (75 MHz, $CDCl_3$)

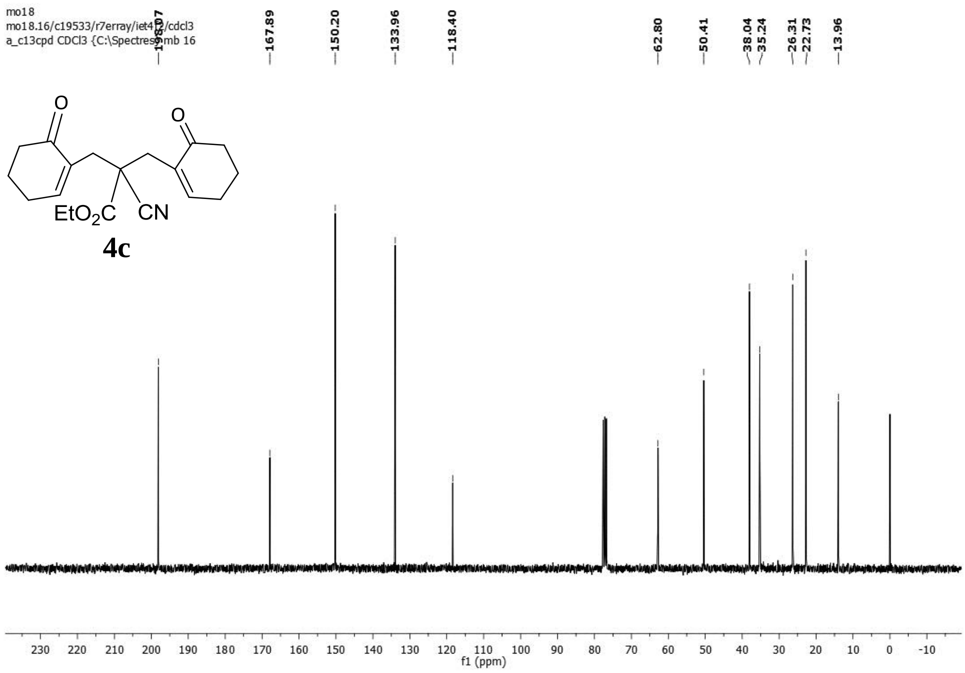

3d ^{1}H NMR (300 MHz, $CDCl_3$)

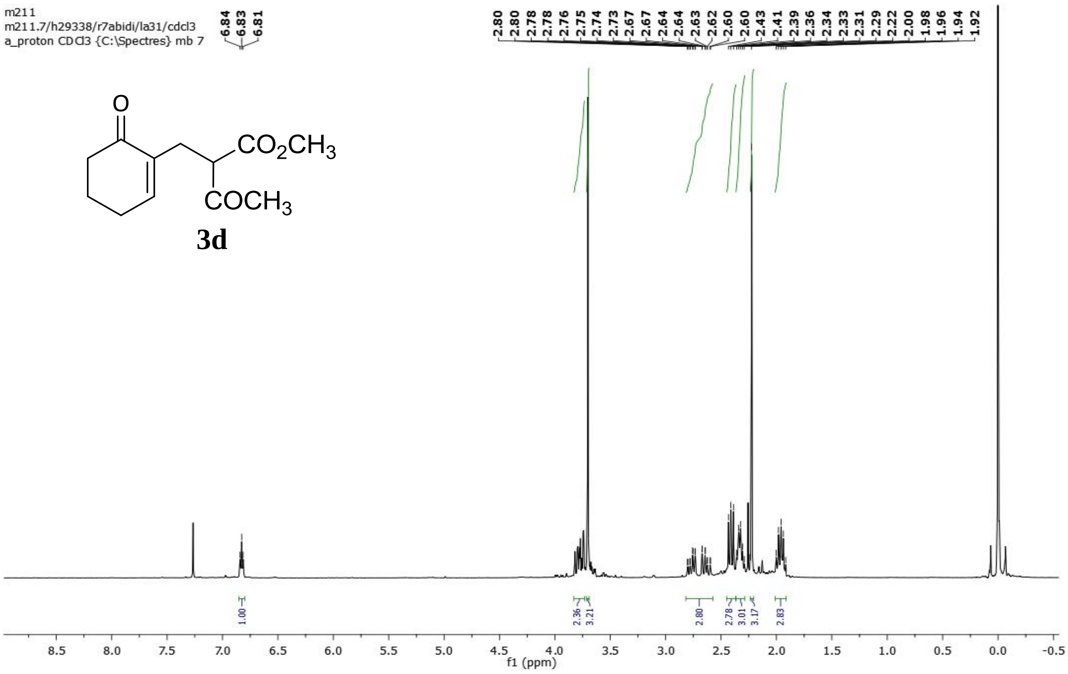

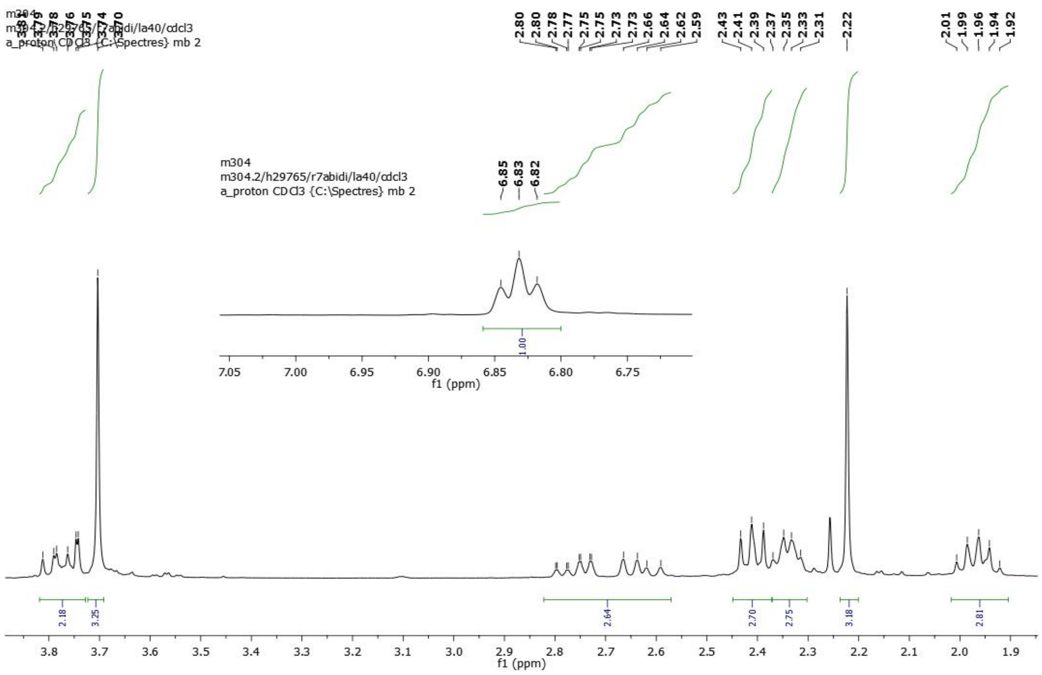

^{13}C NMR (75 MHz, $CDCl_3$)

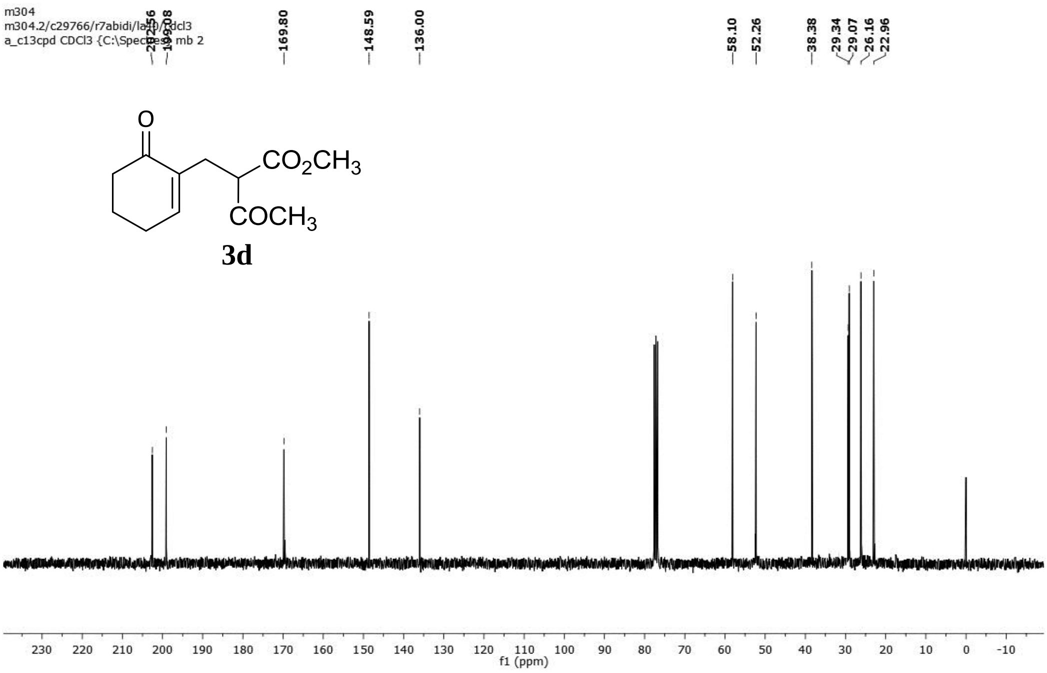

3e ^{1}H NMR (300 MHz, $CDCl_3$)

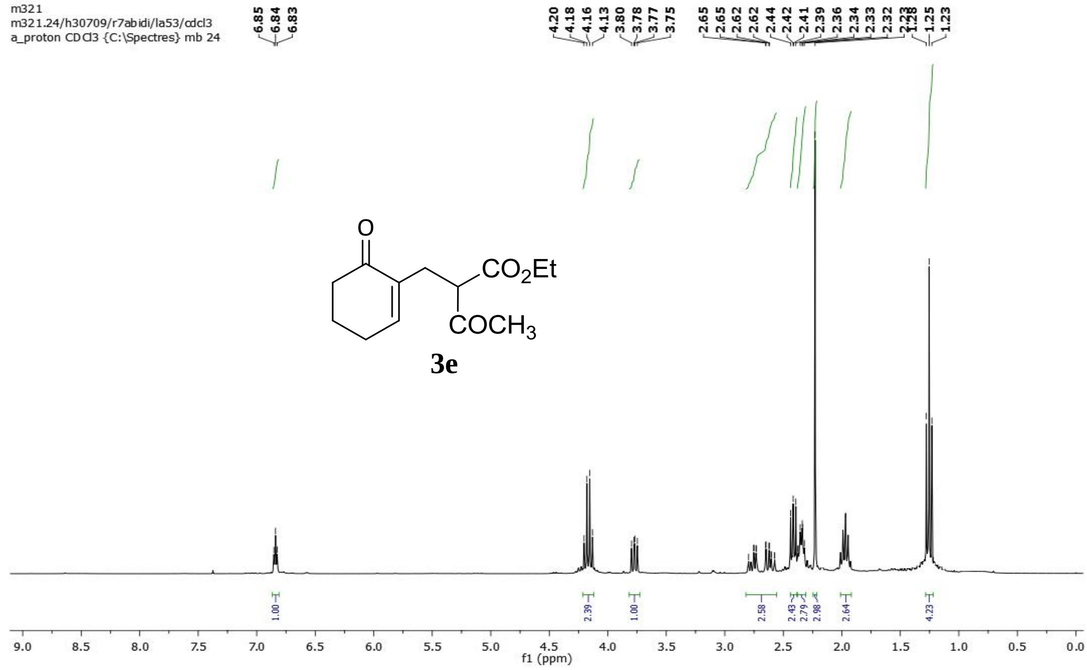

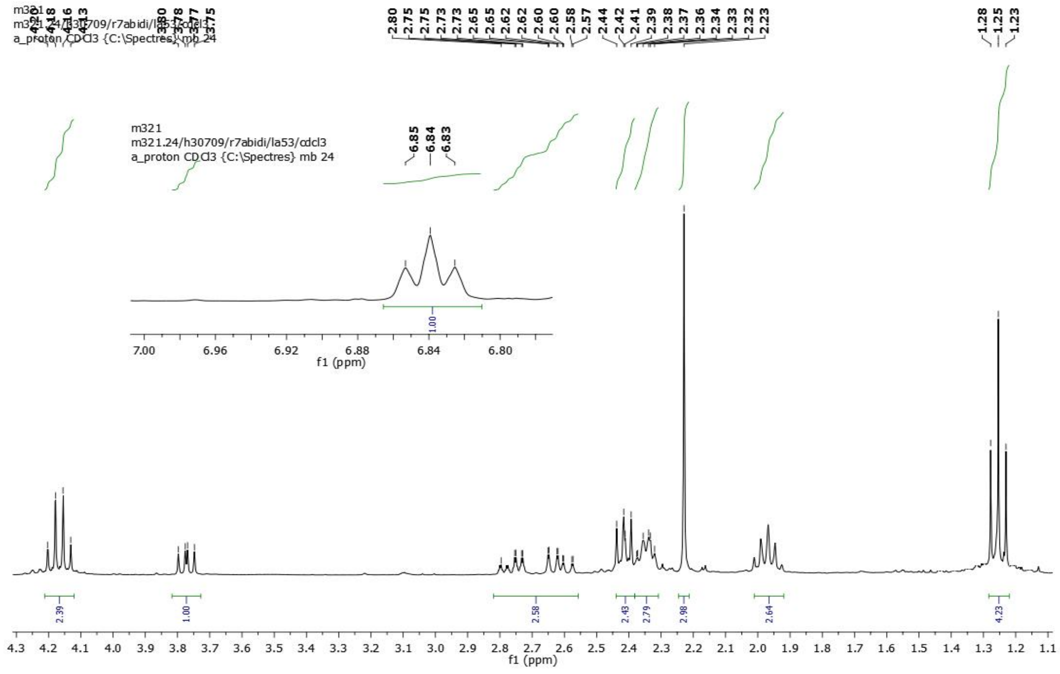

^{13}C NMR (75 MHz, $CDCl_3$)

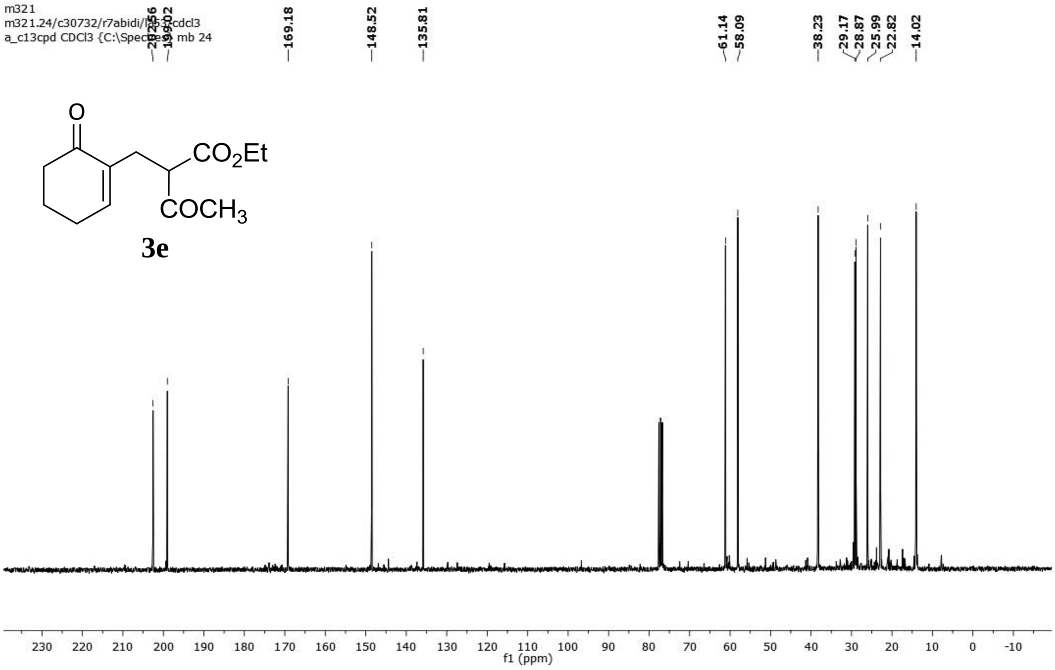

3f ^{1}H NMR (300 MHz, $CDCl_3$)

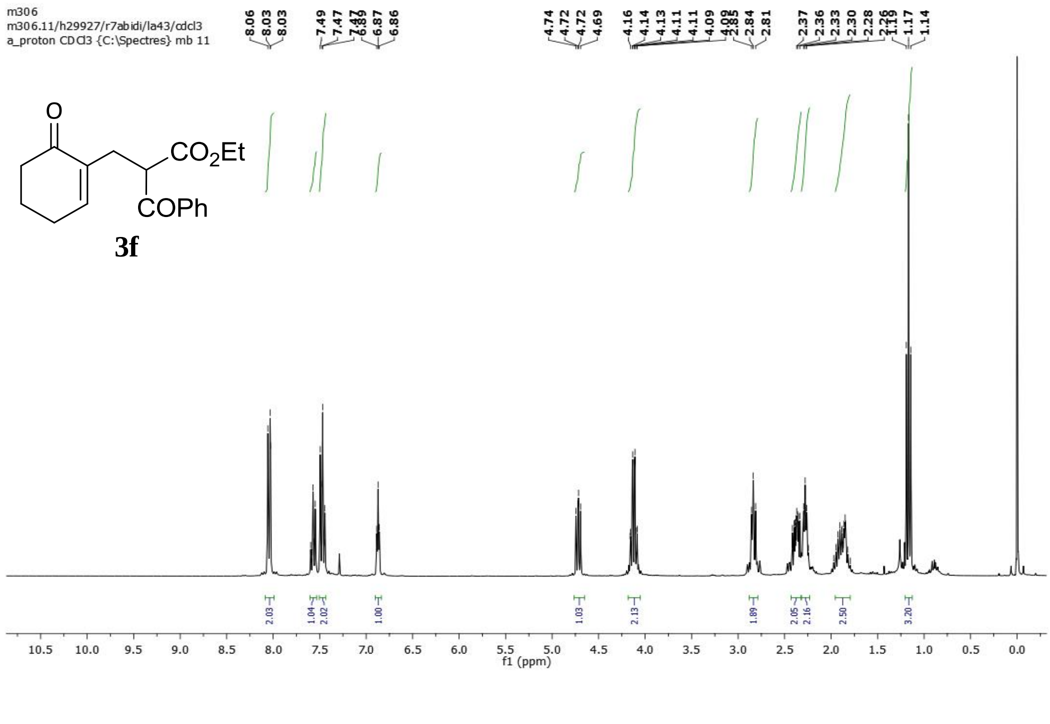

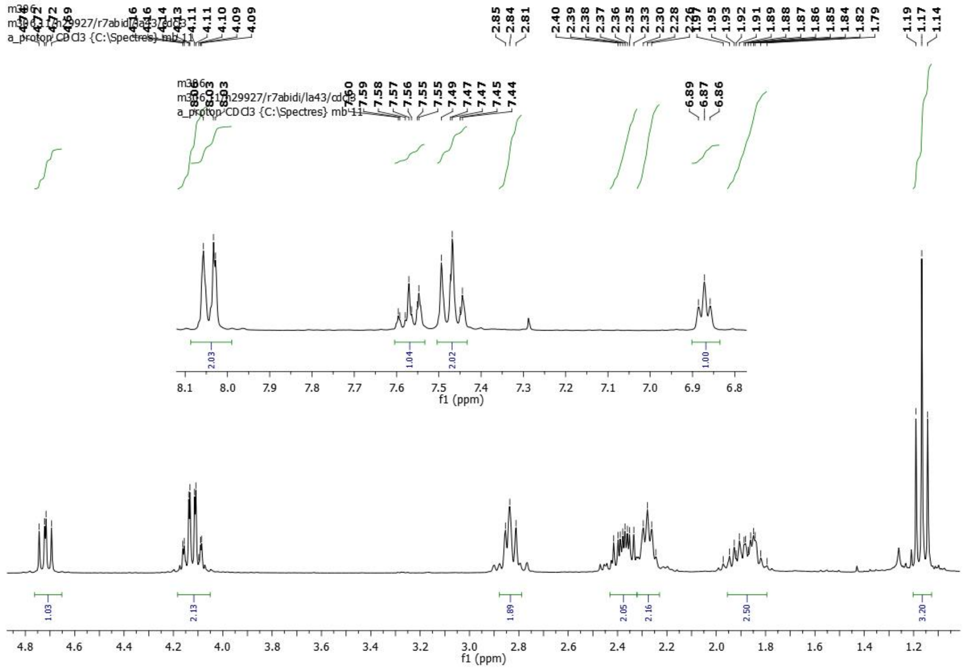

^{13}C NMR (75 MHz, $CDCl_3$)

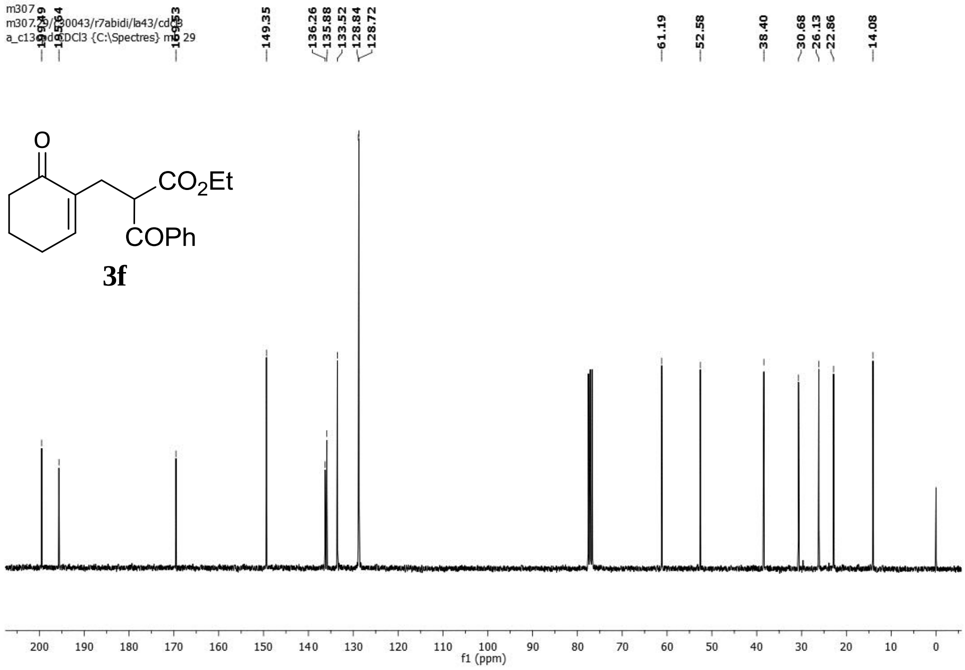

3g ^{1}H NMR (300 MHz, $CDCl_3$)

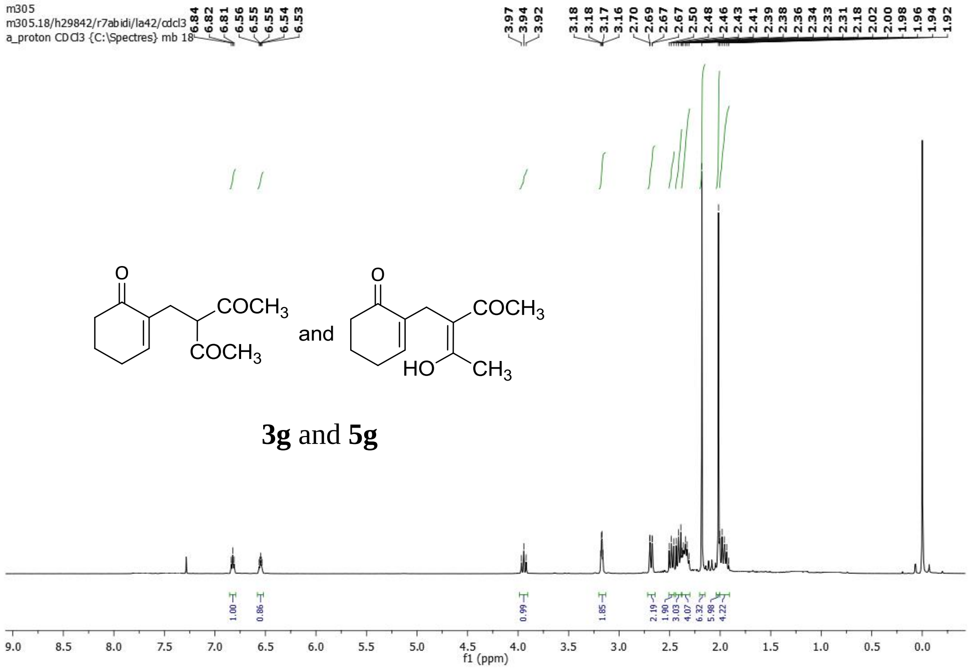

3g and **5g**

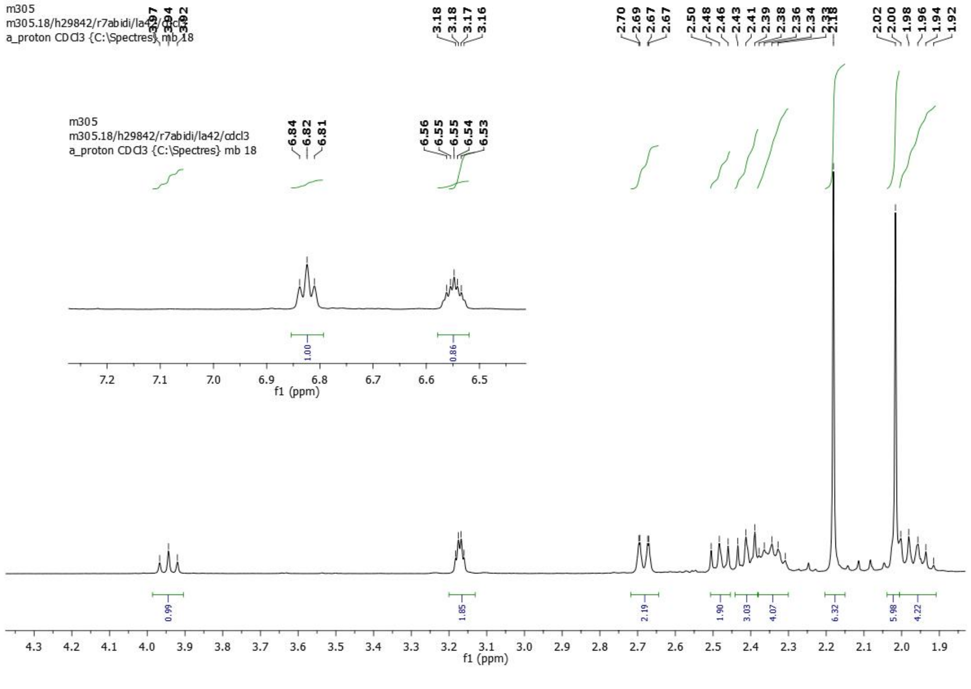

^{13}C NMR (75 MHz, $CDCl_3$)

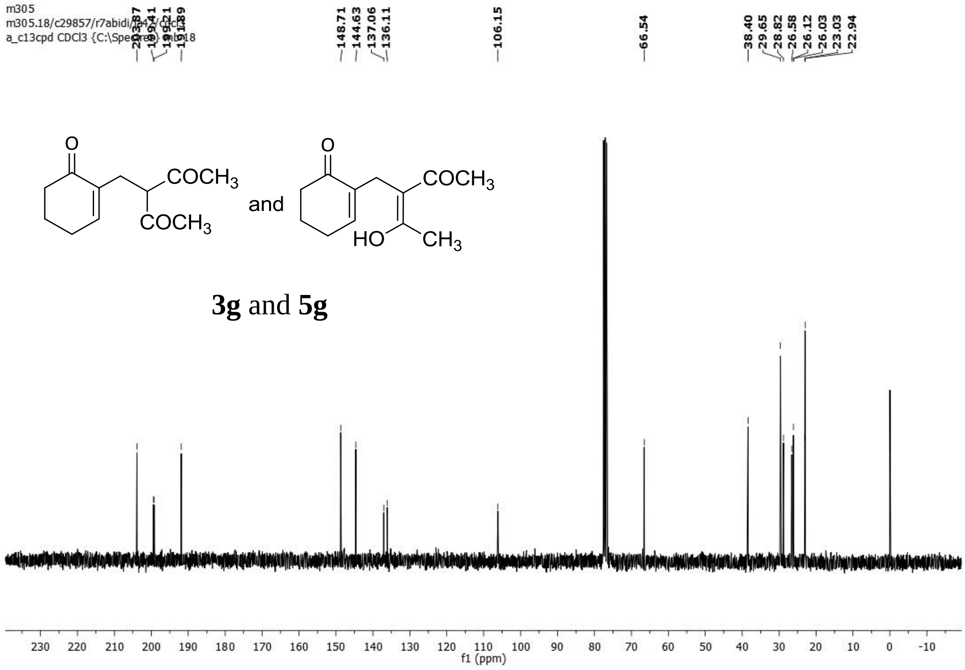

3g and **5g**

3h ^{1}H NMR (300 MHz, $CDCl_3$)

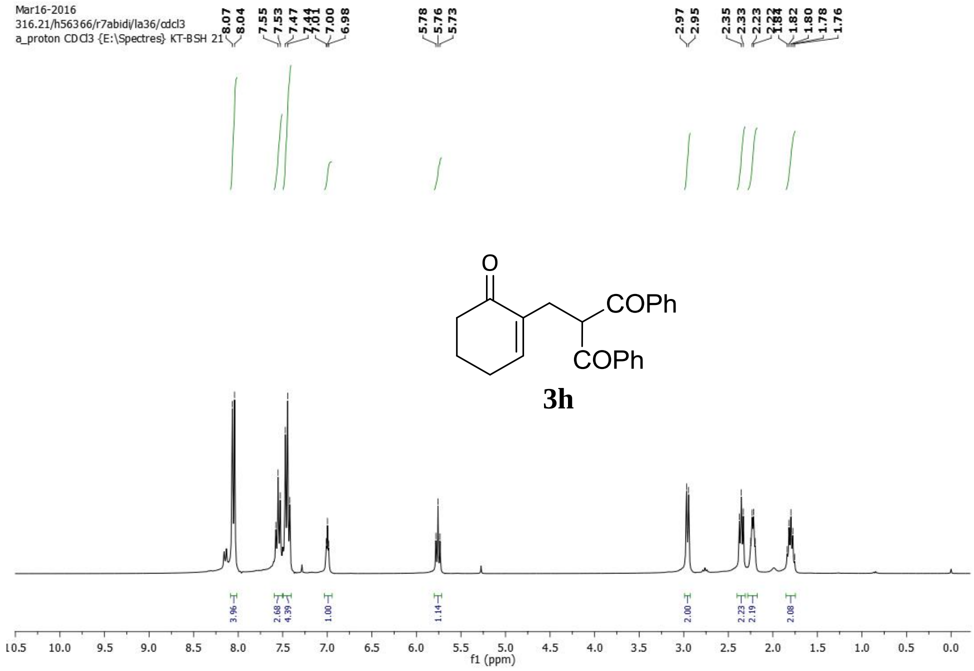

3h

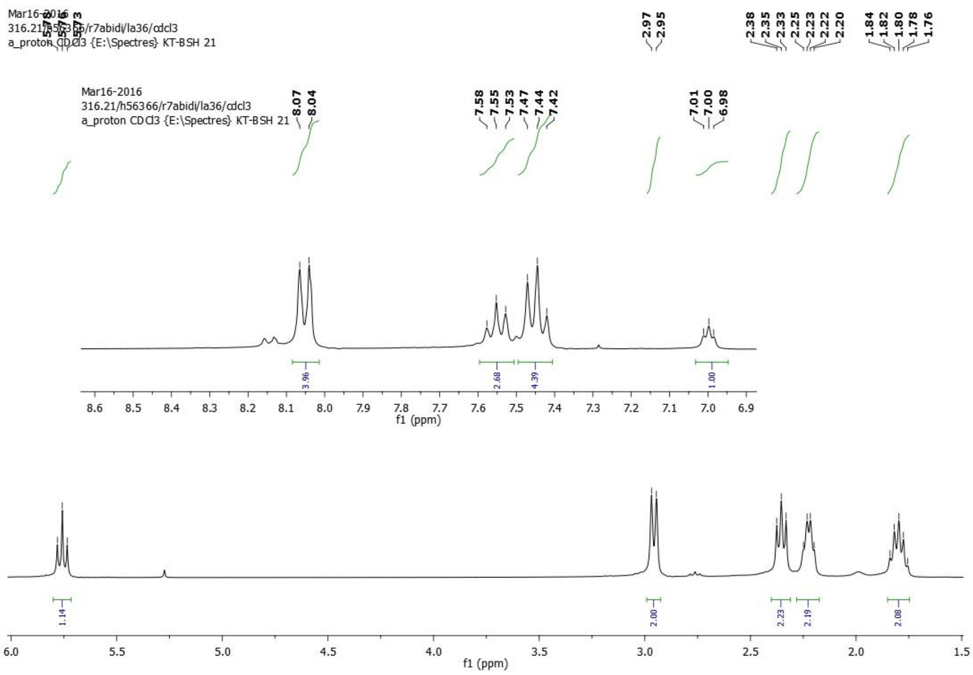

^{13}C NMR (75 MHz, $CDCl_3$)

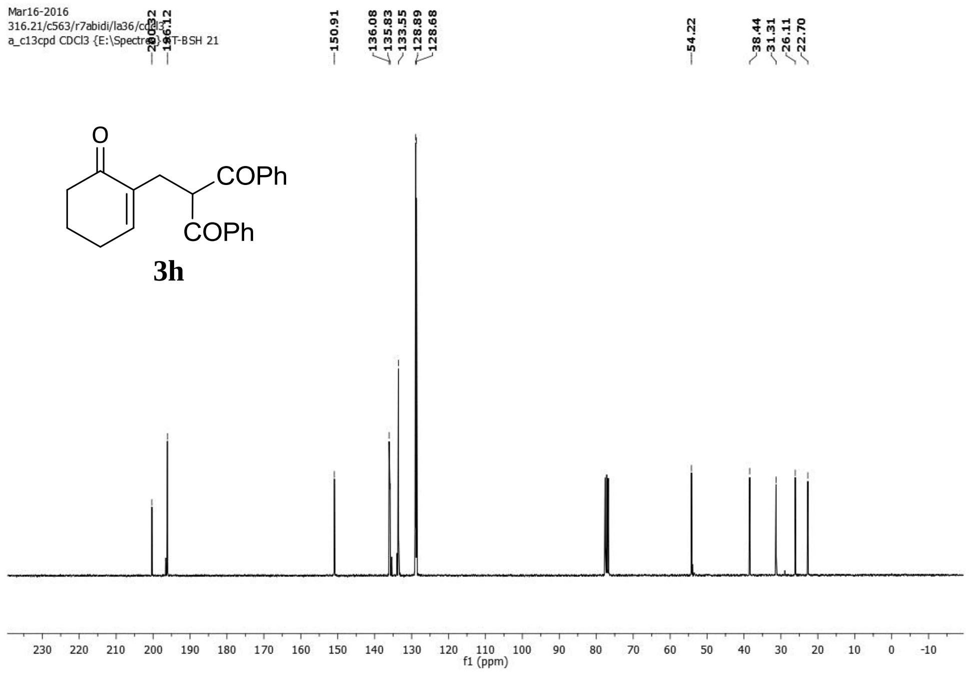

3i ^{1}H NMR (300 MHz, $CDCl_3$)

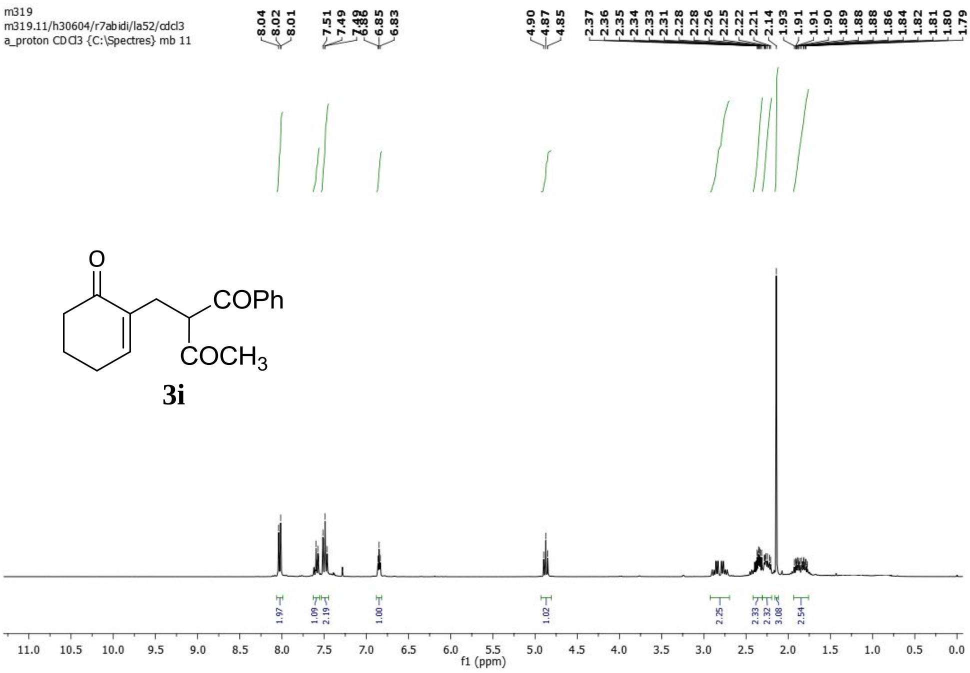

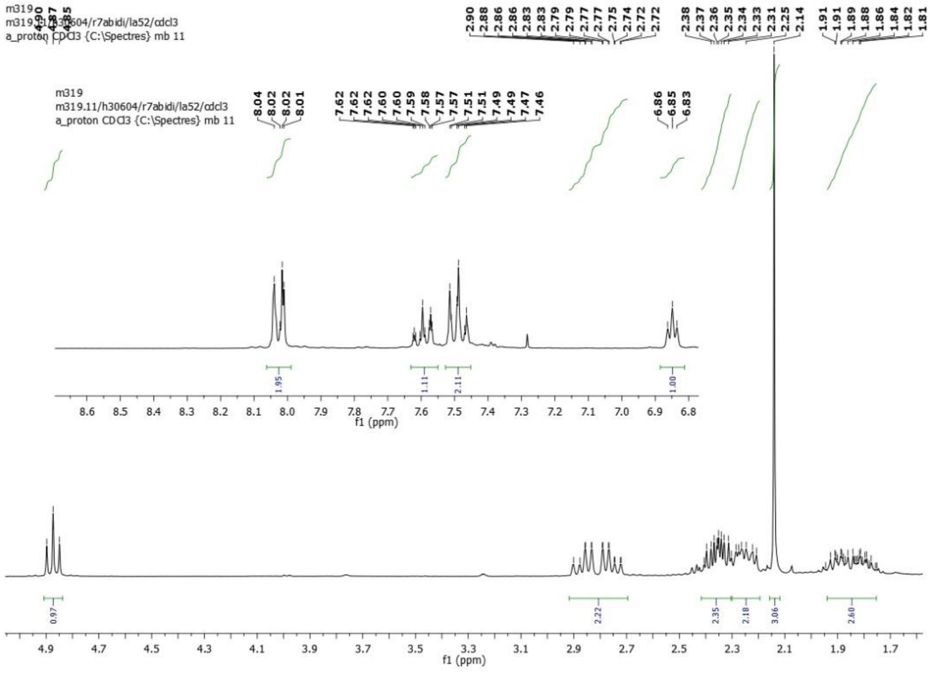

^{13}C NMR (75 MHz, $CDCl_3$)

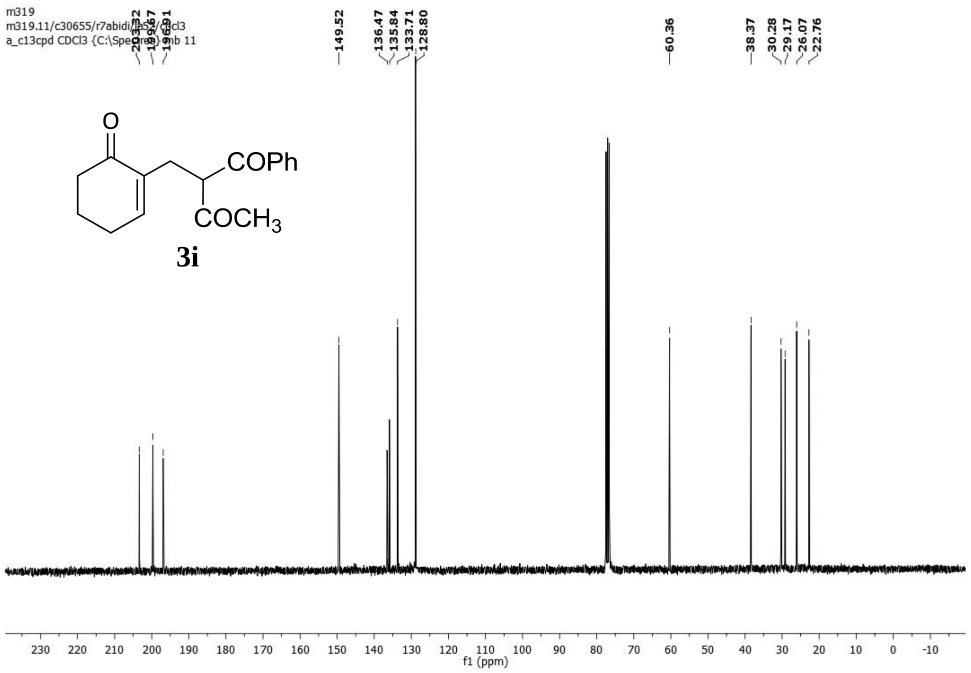

3j ^{1}H NMR (300 MHz, $CDCl_3$)

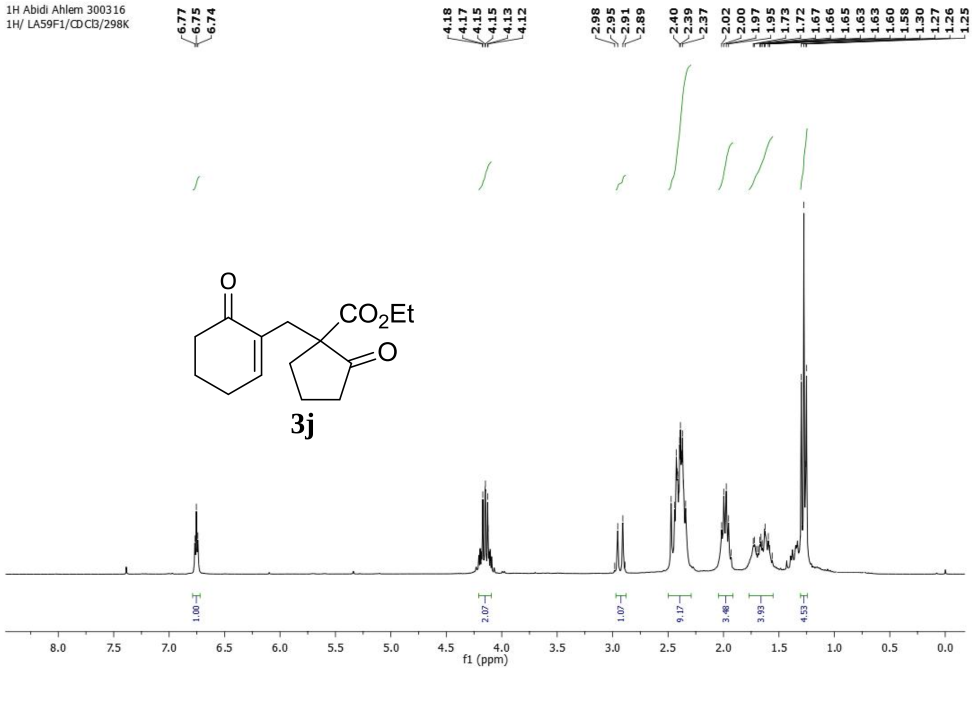

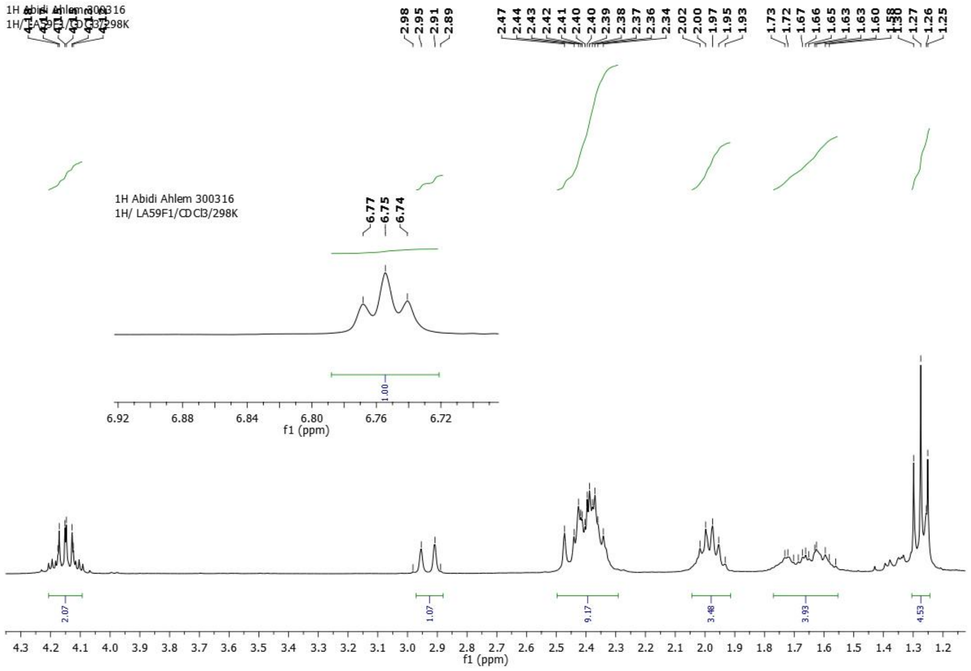

^{13}C NMR (75 MHz, $CDCl_3$)

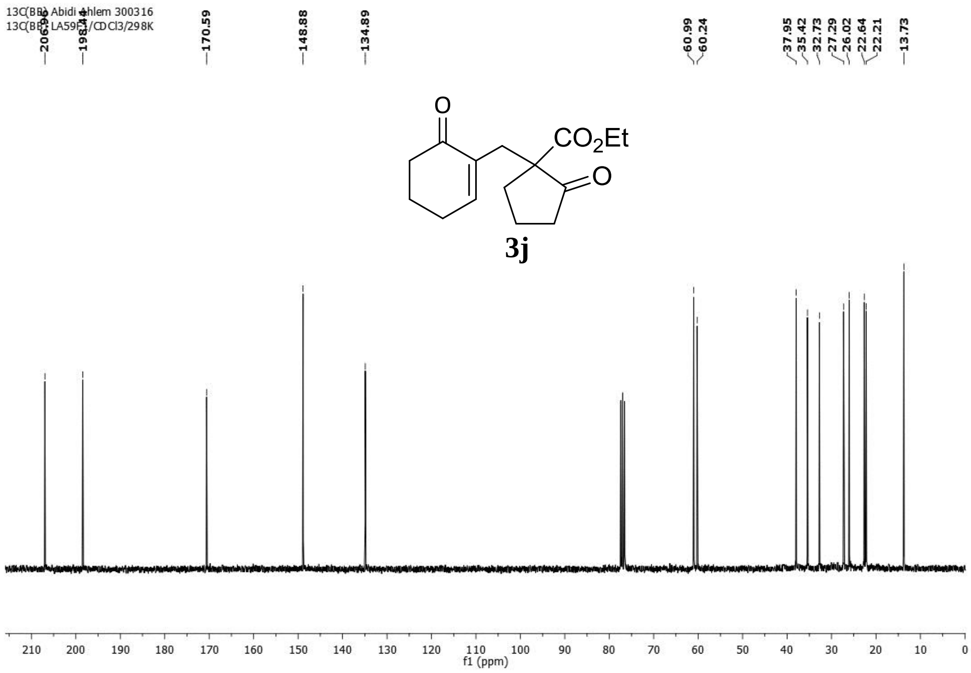

6j ^{1}H NMR (300 MHz, $CDCl_3$)

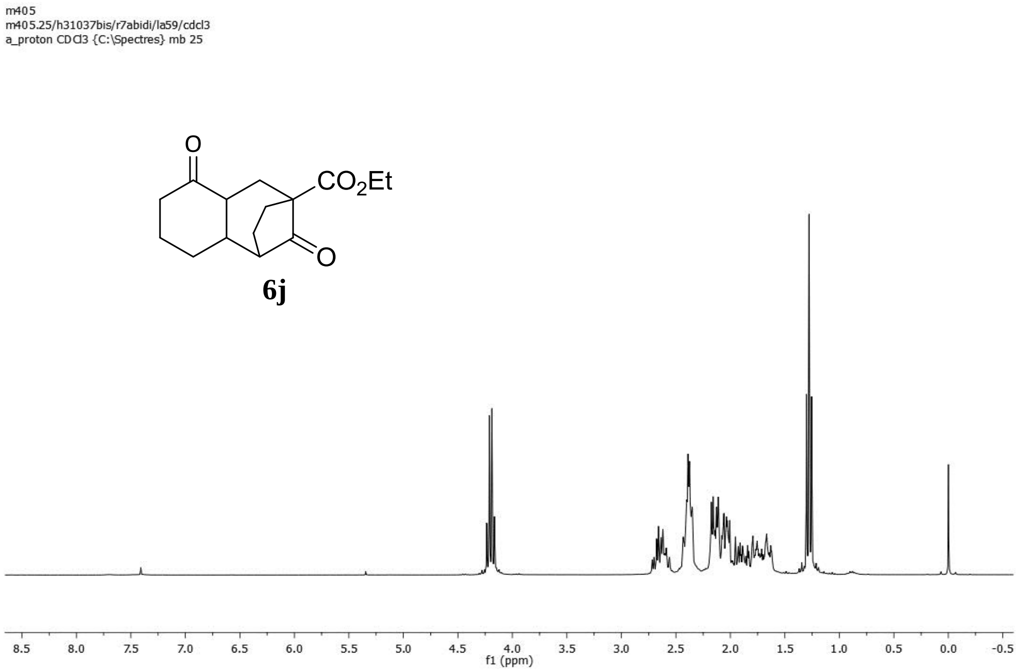

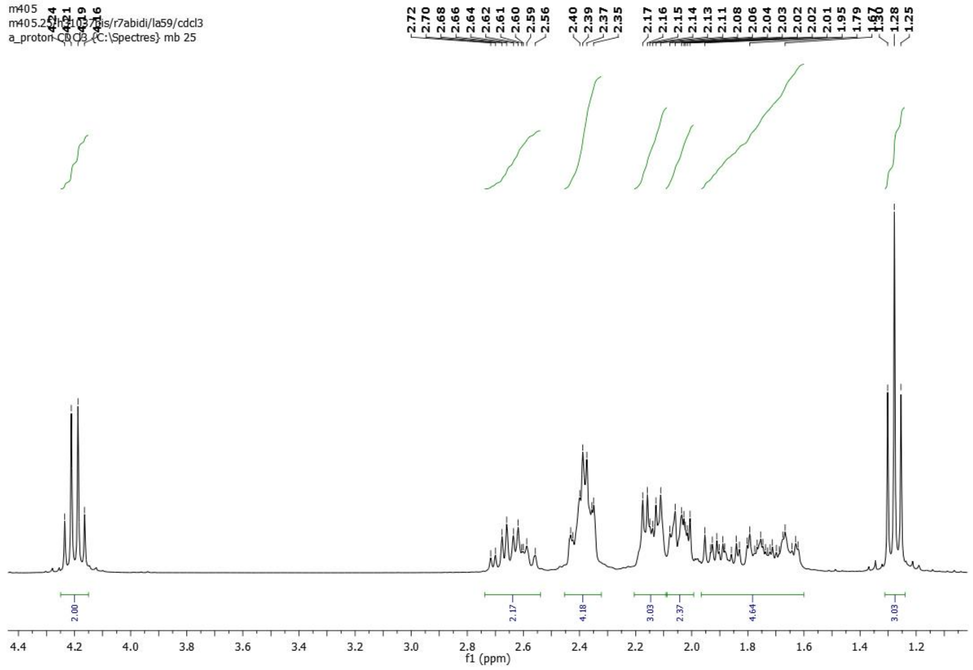

^{13}C NMR (75 MHz, $CDCl_3$)

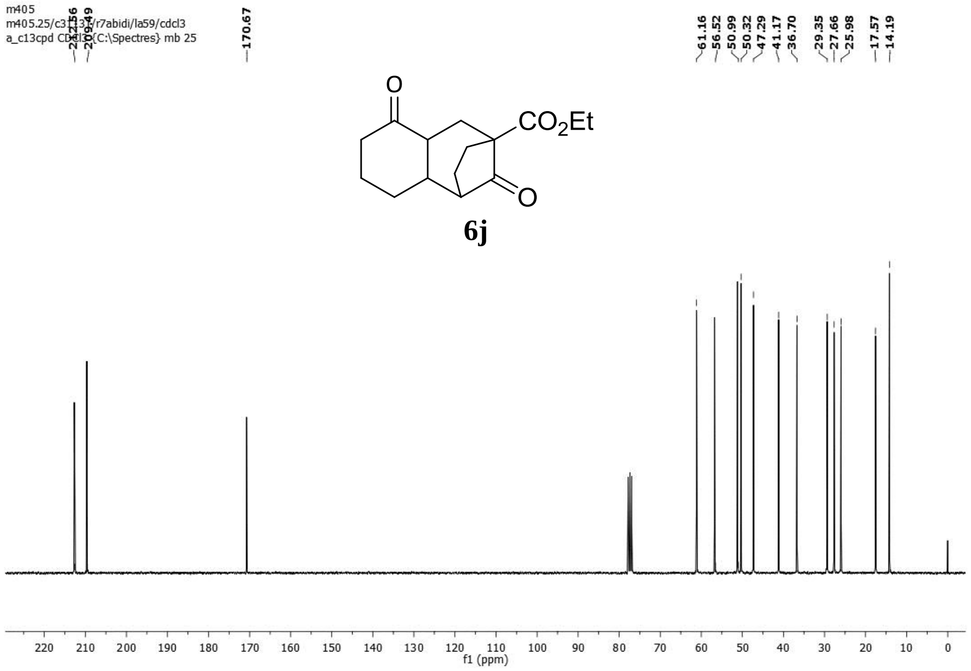

7e ^{1}H NMR (300 MHz, $CDCl_3$)

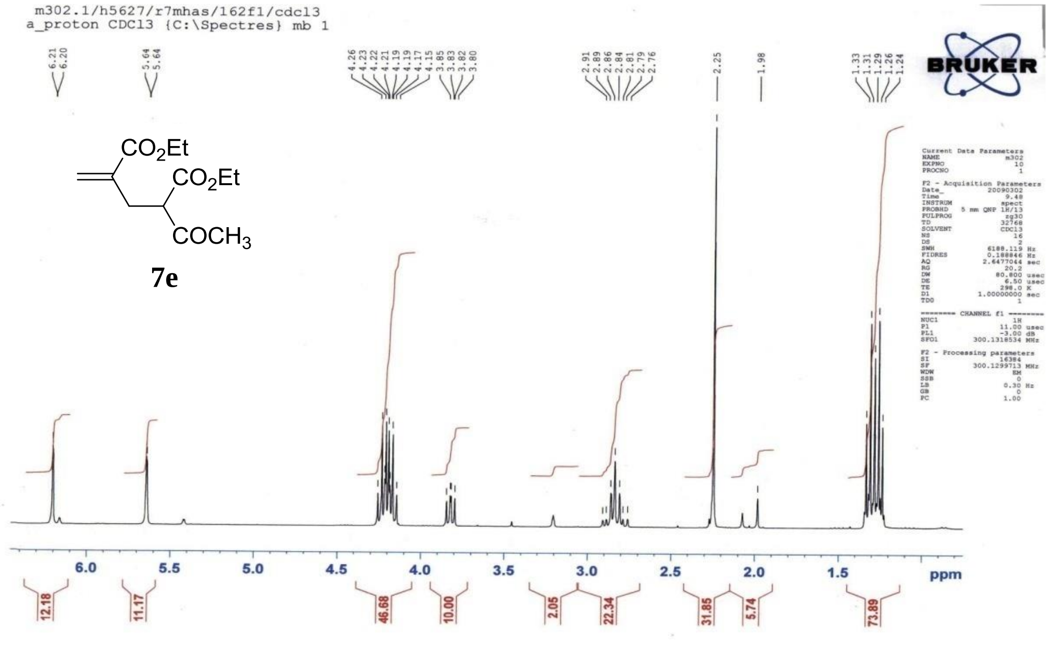

7e ^{13}C NMR (75 MHz, $CDCl_3$)

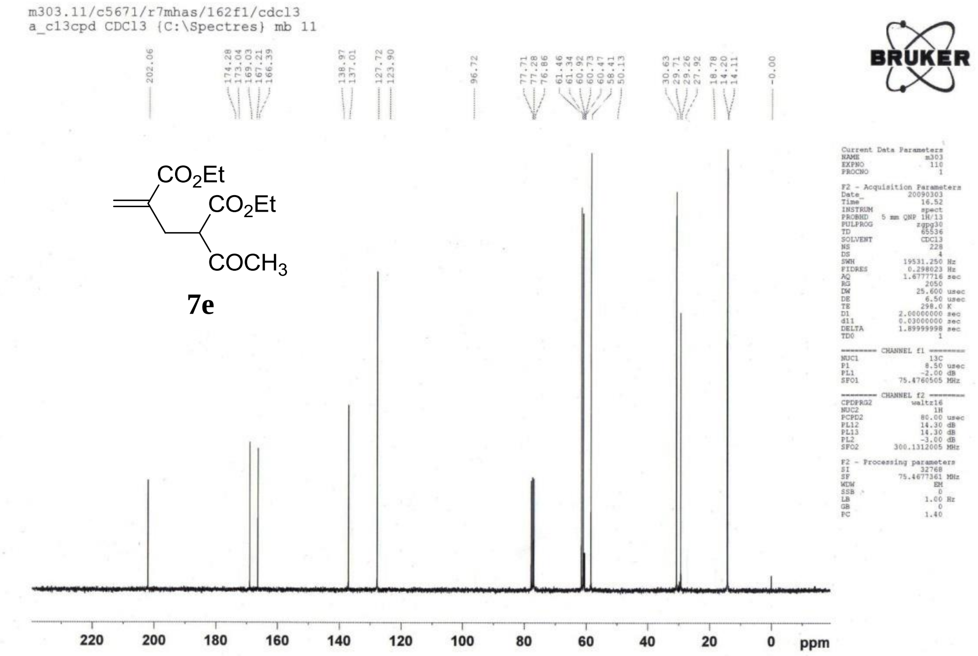

7f ^{1}H NMR (300 MHz, $CDCl_3$)

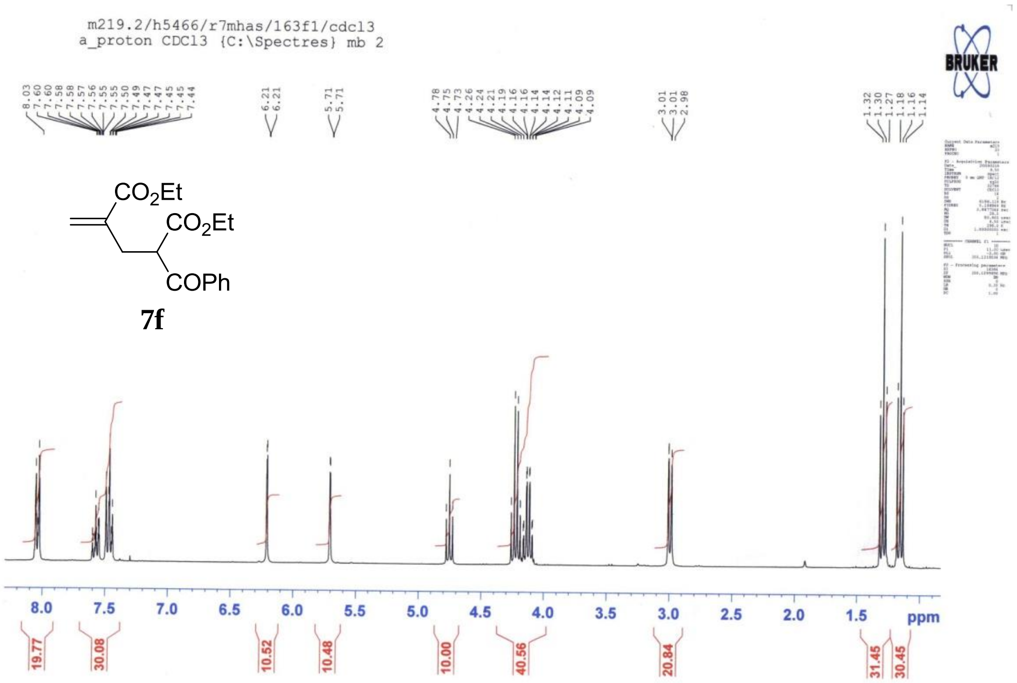

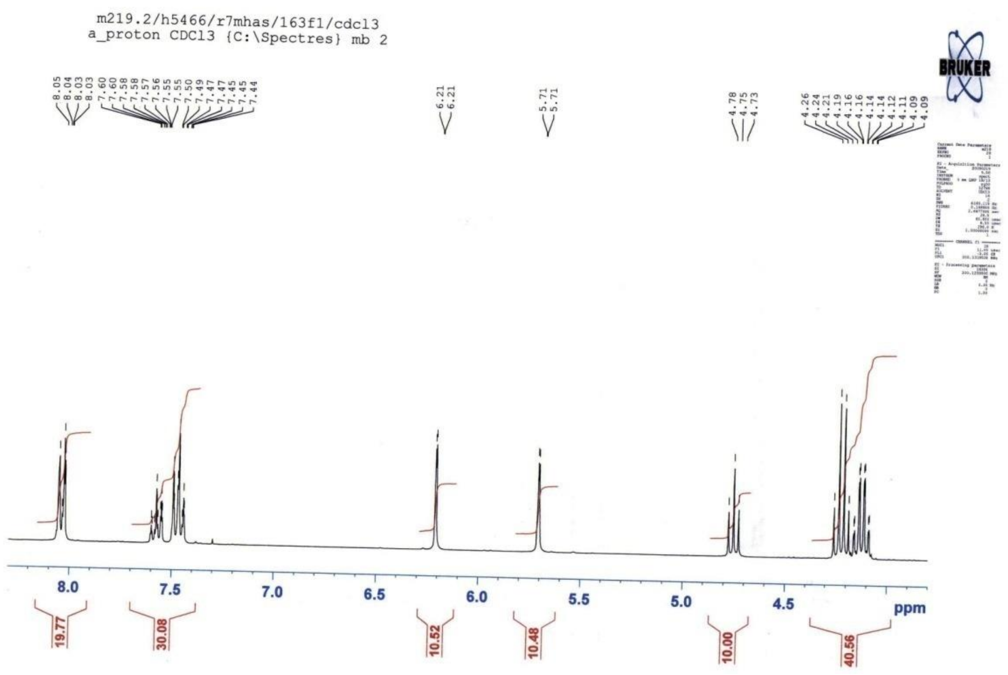

7f ^{13}C NMR (75 MHz, $CDCl_3$)

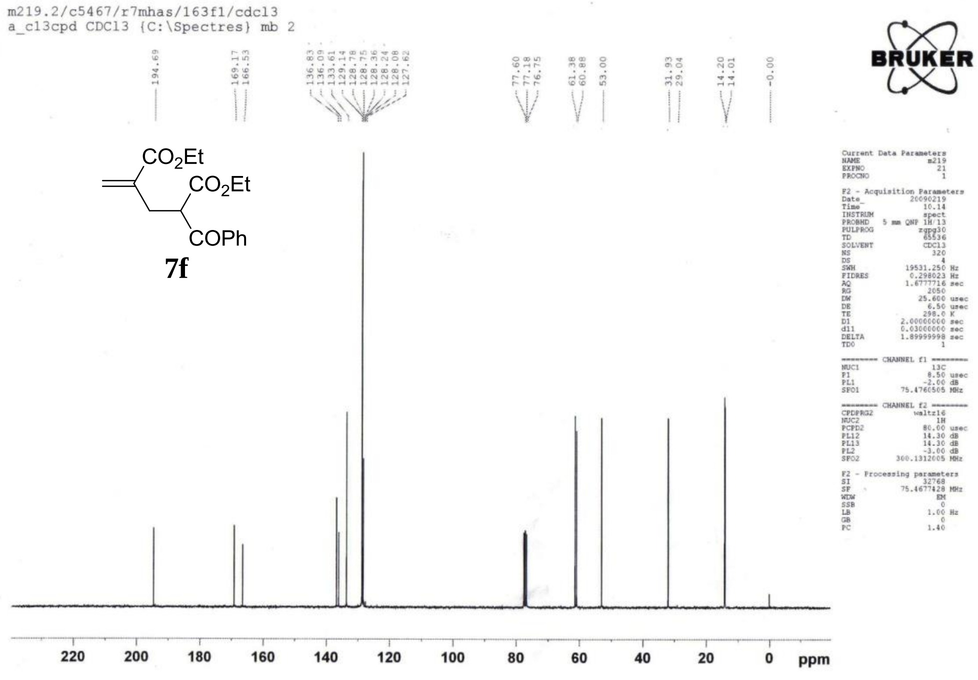

7g and **8g** ^{1}H NMR (300 MHz, $CDCl_3$)

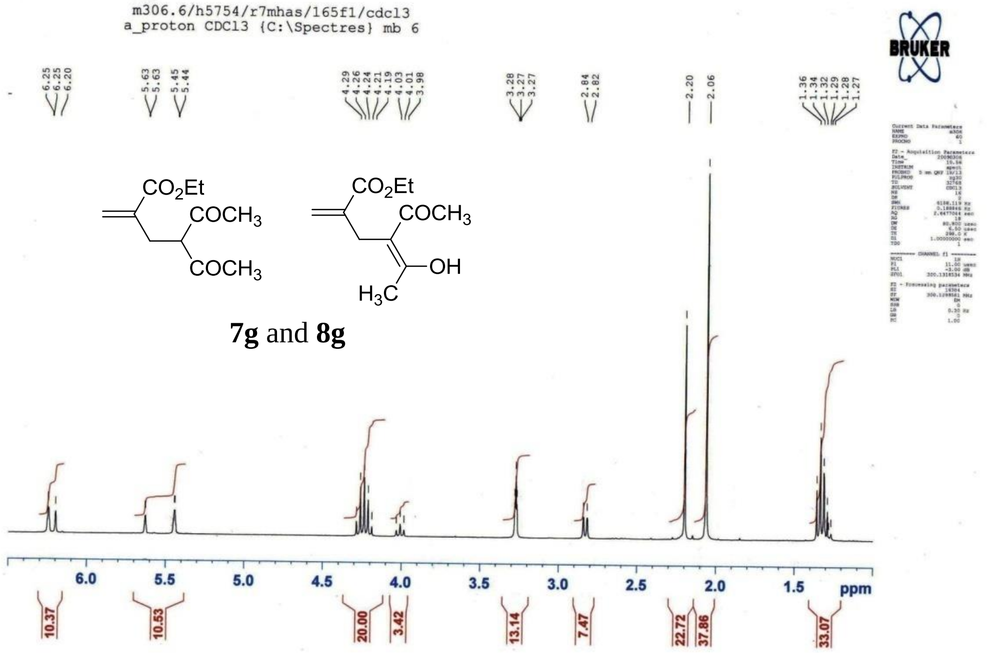

7g and **8g**^{13}C NMR (75 MHz, $CDCl_3$)

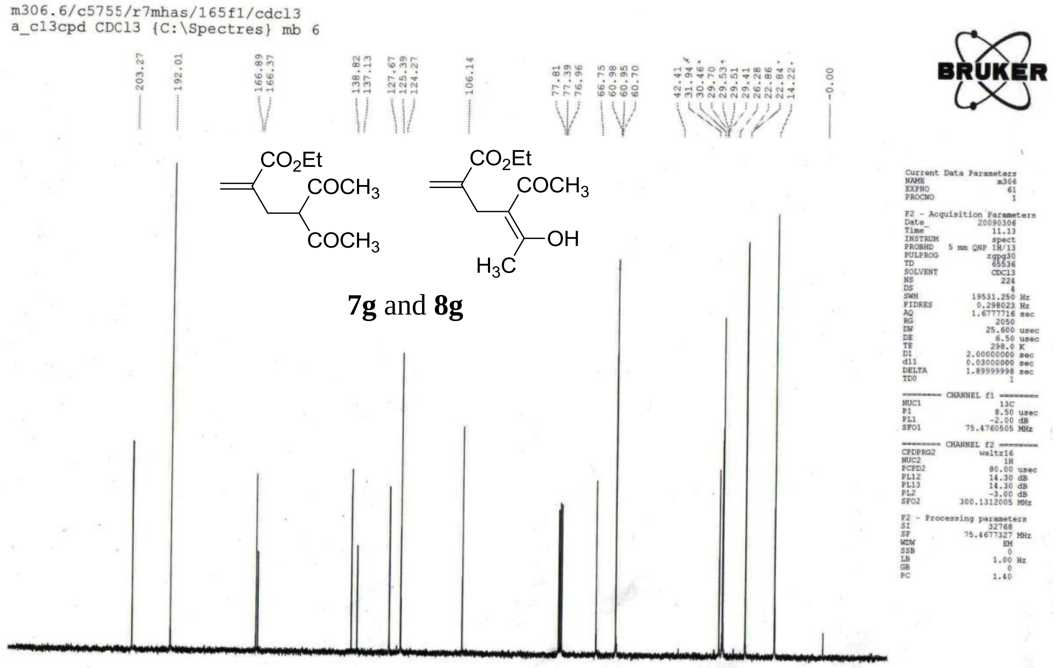

7i ^{1}H NMR (300 MHz, $CDCl_3$)

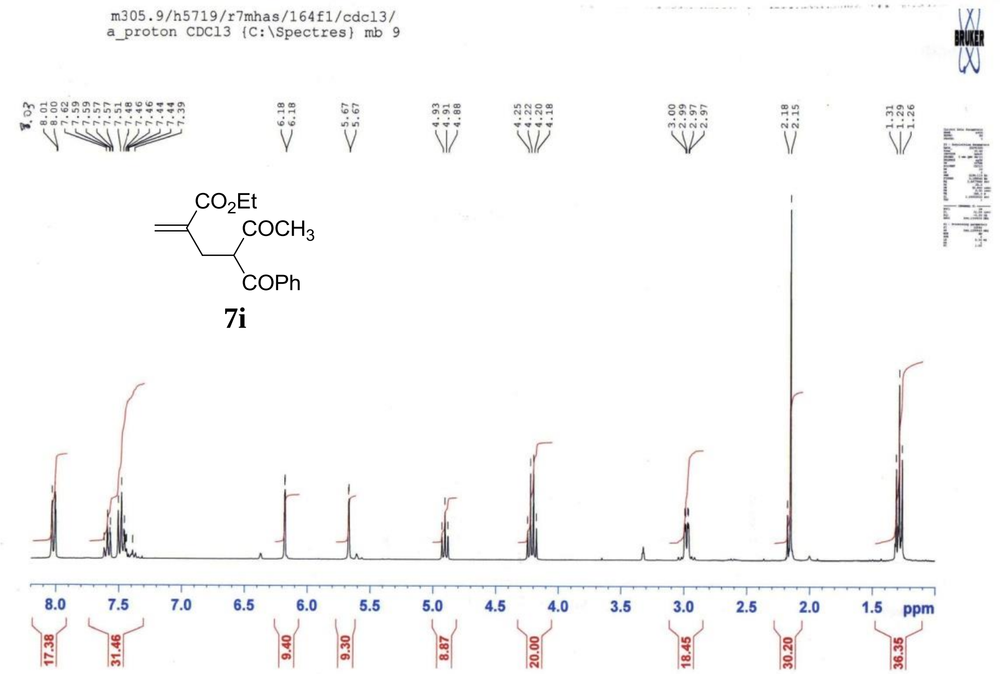

7i ^{13}C NMR (75 MHz, $CDCl_3$)

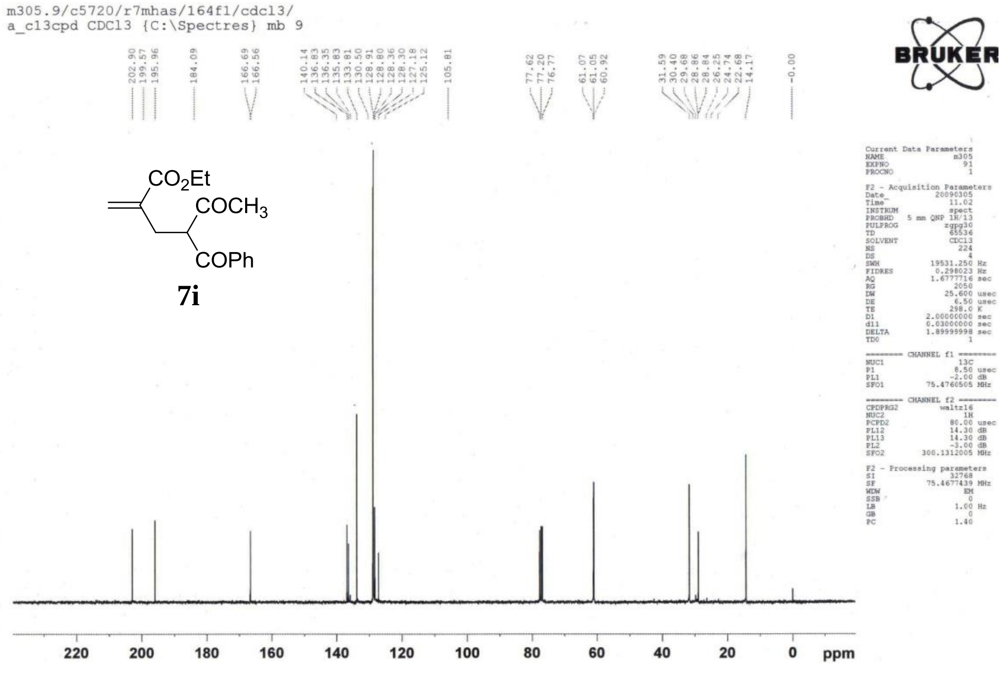

GC-MS data for compounds **3a-7i**

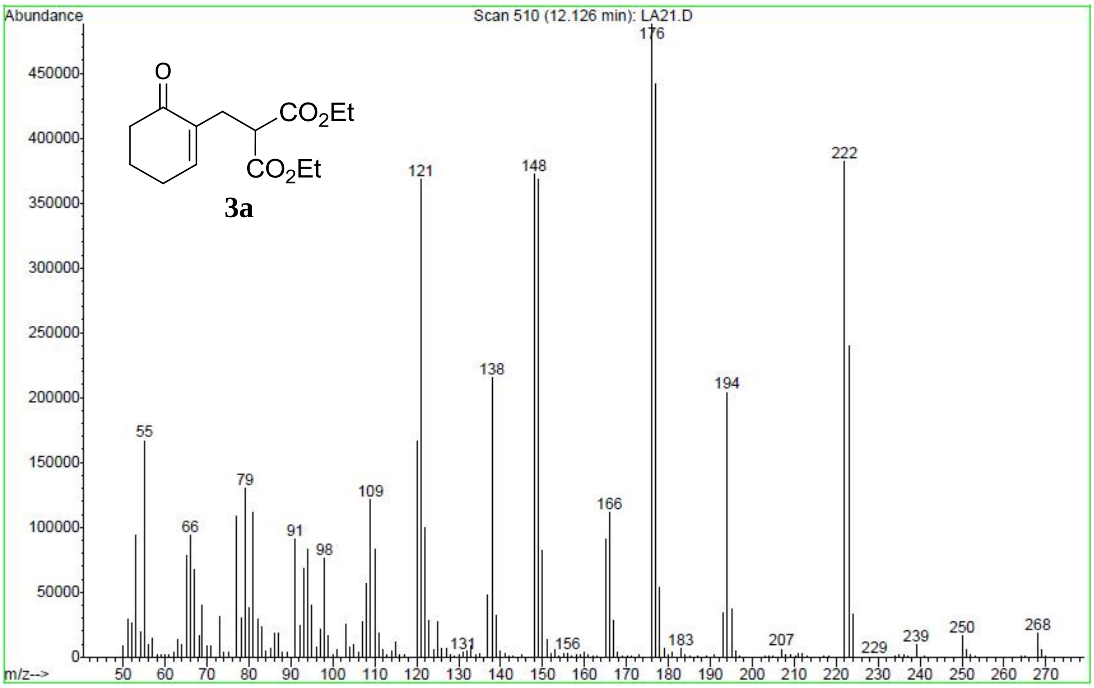

3a

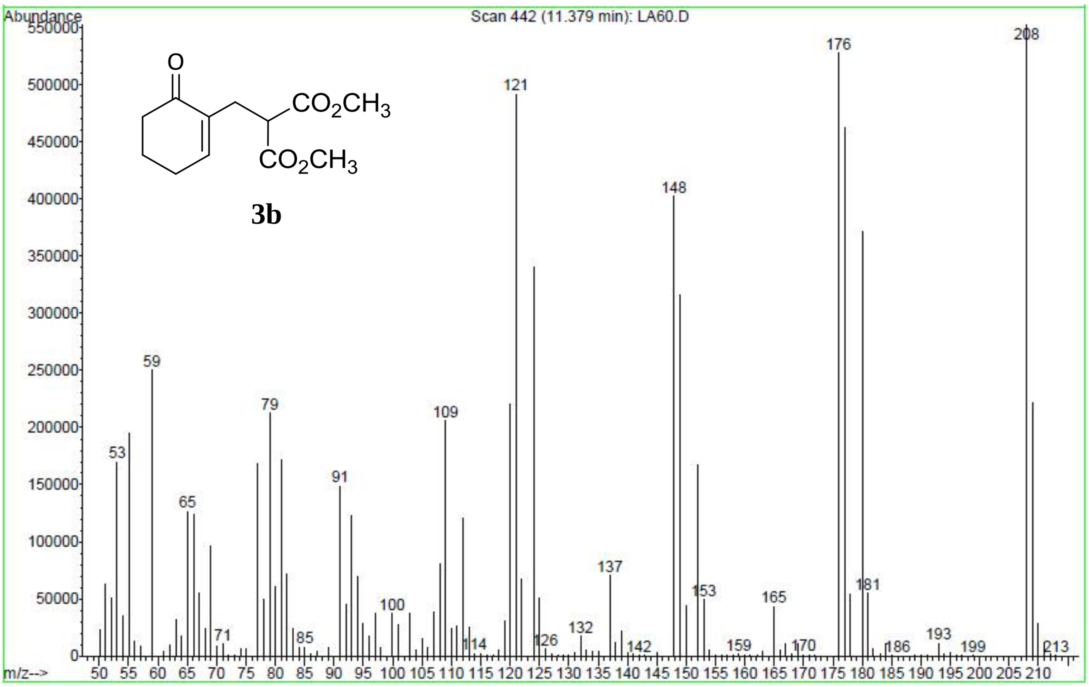

3b

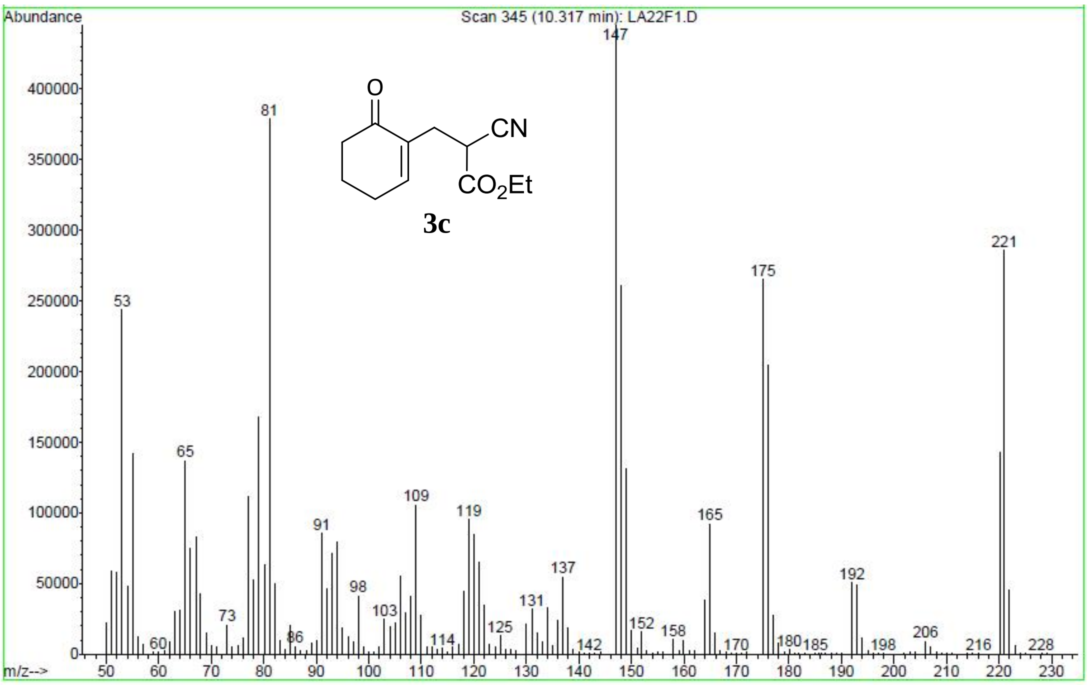
Abundance
Scan 345 (10.317 min): LA22F1.D
CN
CO2Et
3c
m/z-->

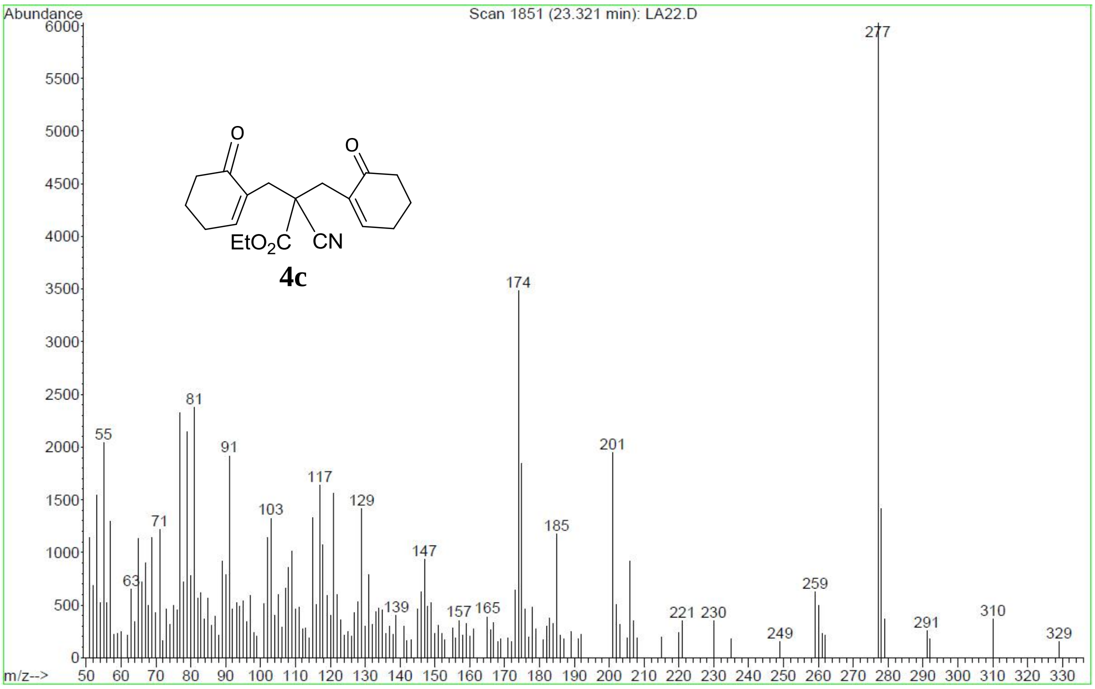
Abundance
Scan 1851 (23.321 min): LA22.D
EtO2C
CN
4c
m/z-->

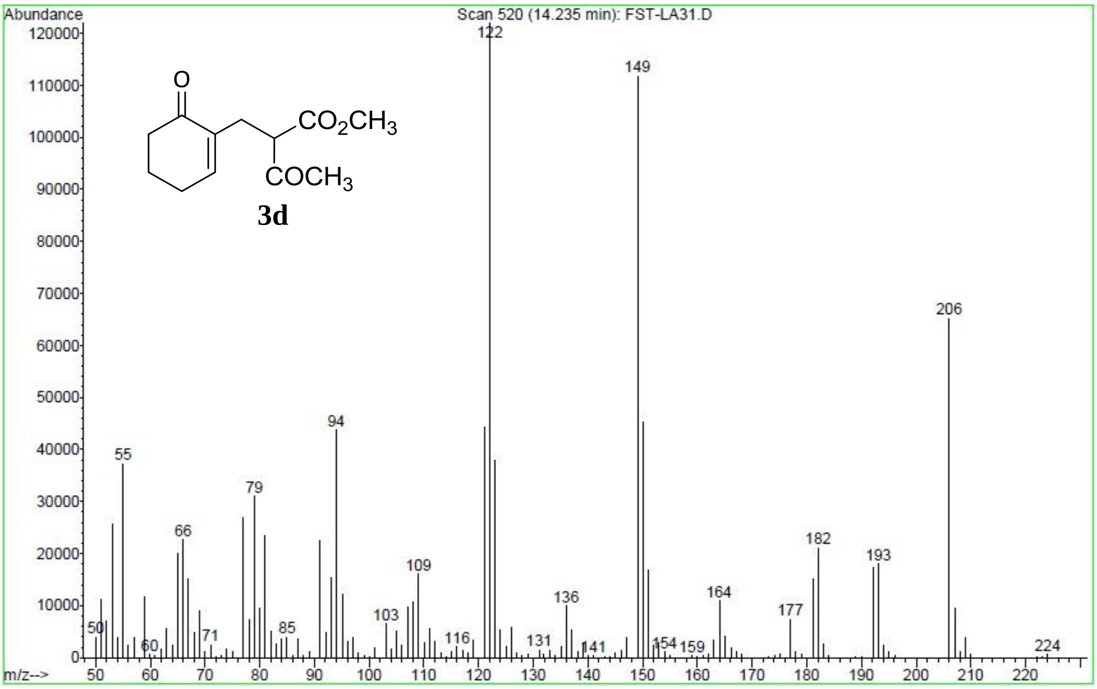
Abundance
Scan 520 (14.235 min): FST-LA31.D
CO_2CH_3
$COCH_3$
O
3d
m/z-->
122
149
206
94
55
79
66
182
193
109
164
136
177
103
85
50
71
116
131
141
154
159
60
224

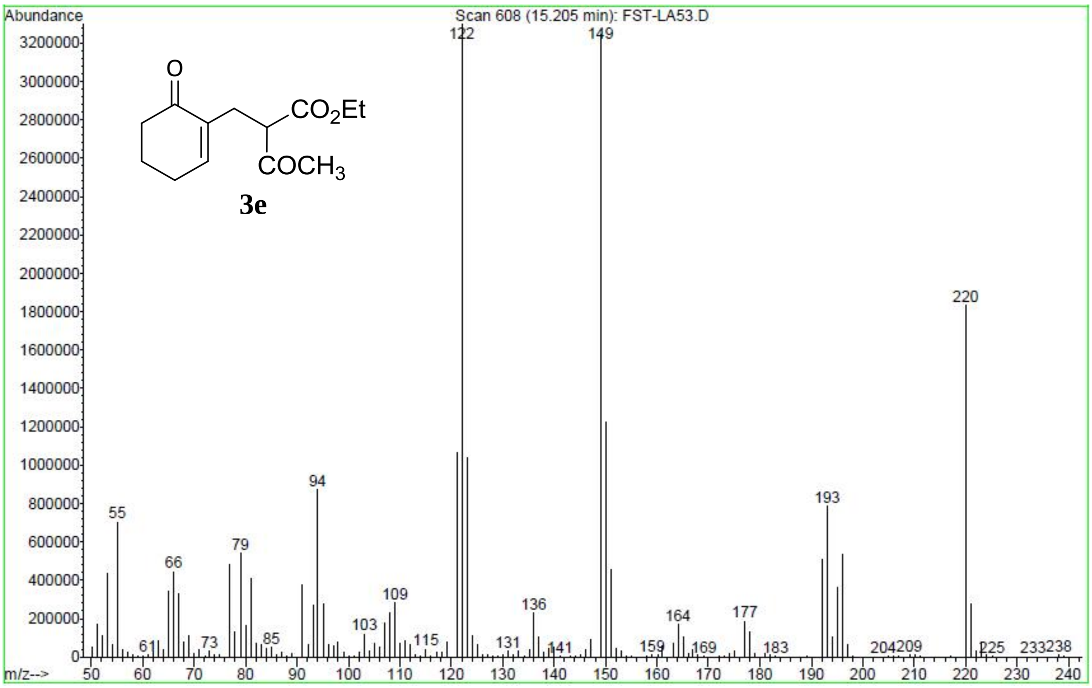
Abundance
Scan 608 (15.205 min): FST-LA53.D
CO_2Et
$COCH_3$
O
3e
m/z-->
122
149
220
94
55
193
79
66
109
136
177
164
103
85
73
61
115
131
141
159
169
183
204209
225
233238

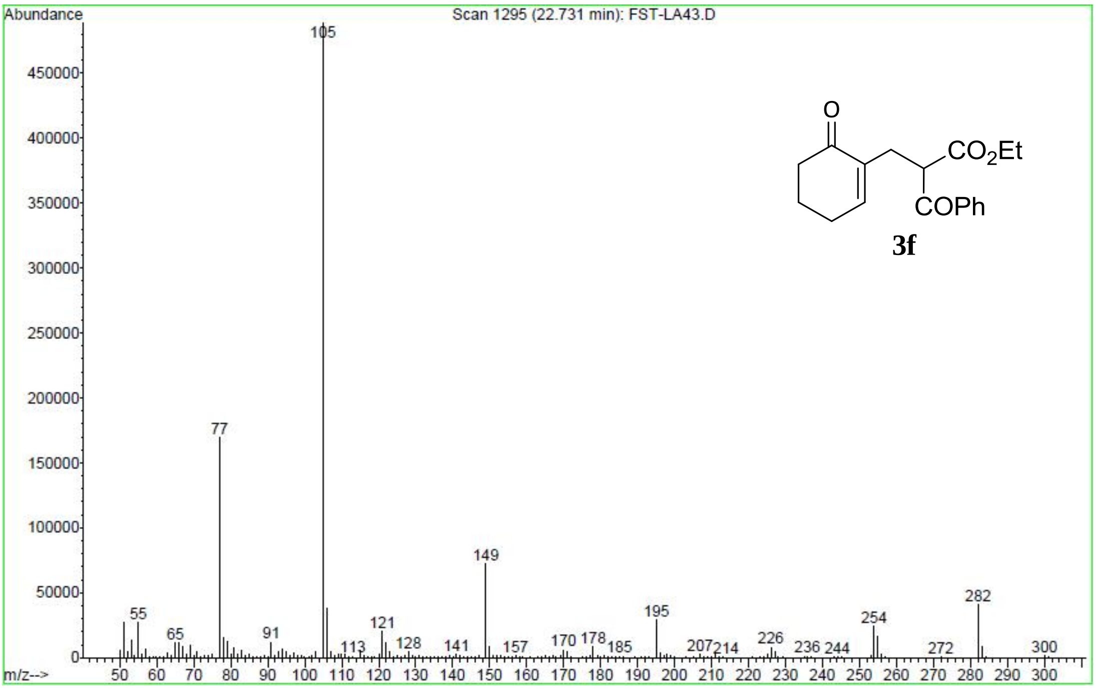
Abundance
Scan 1295 (22.731 min): FST-LA43.D
m/z-->
105
77
149
55
65
91
121
128
113
141
157
170
178
185
195
207
214
226
236
244
254
272
282
300
CO2Et
COPh
O
3f

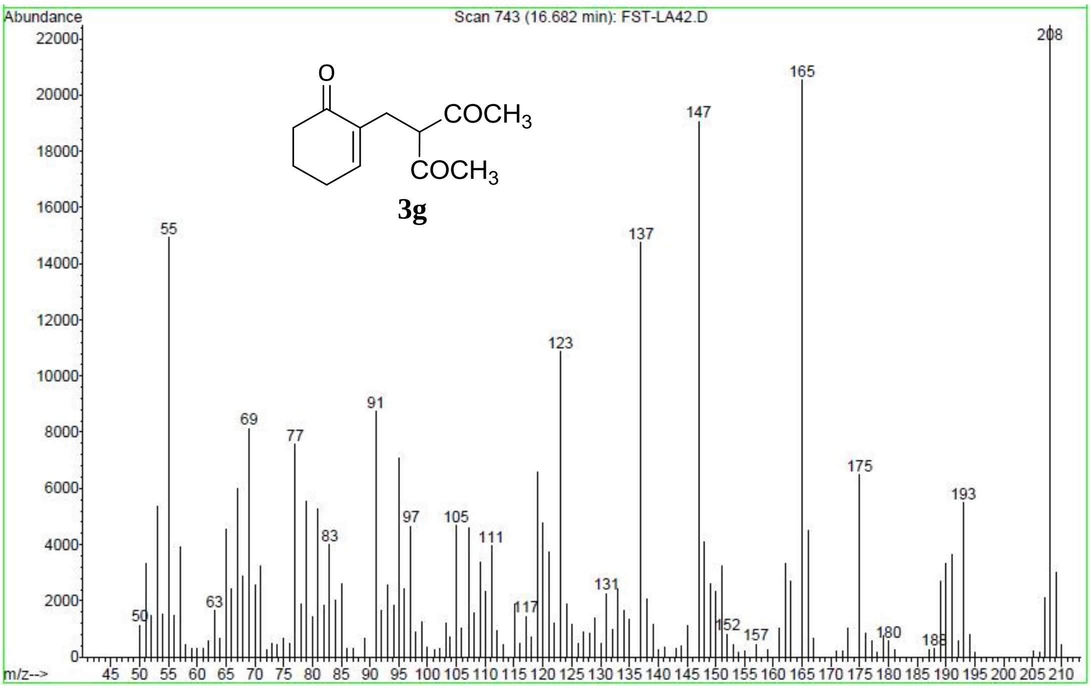
Abundance
Scan 743 (16.682 min): FST-LA42.D
m/z-->
208
165
147
137
55
123
69
77
91
97
105
111
83
63
50
131
117
152
157
175
180
193
COCH3
COCH3
O
3g

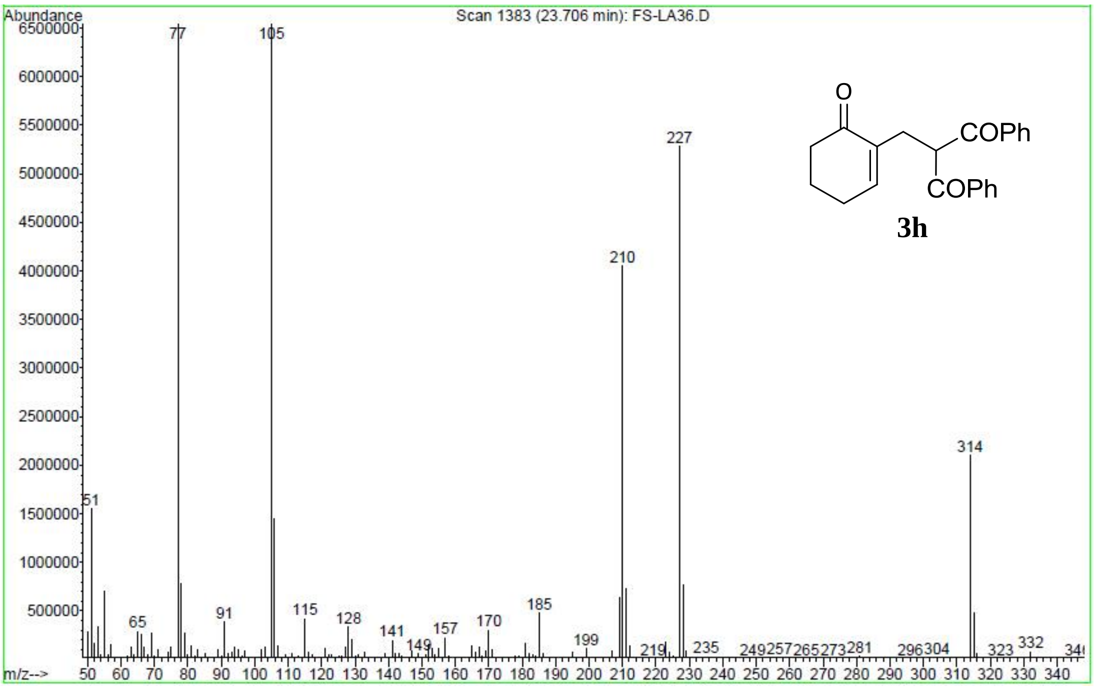
Abundance
Scan 1383 (23.706 min): FS-LA36.D
COPh
COPh
3h
77
105
227
210
314
51
65
91
115
128
141
149
157
170
185
199
219
235
249257265273281
296304
323
332
m/z-->

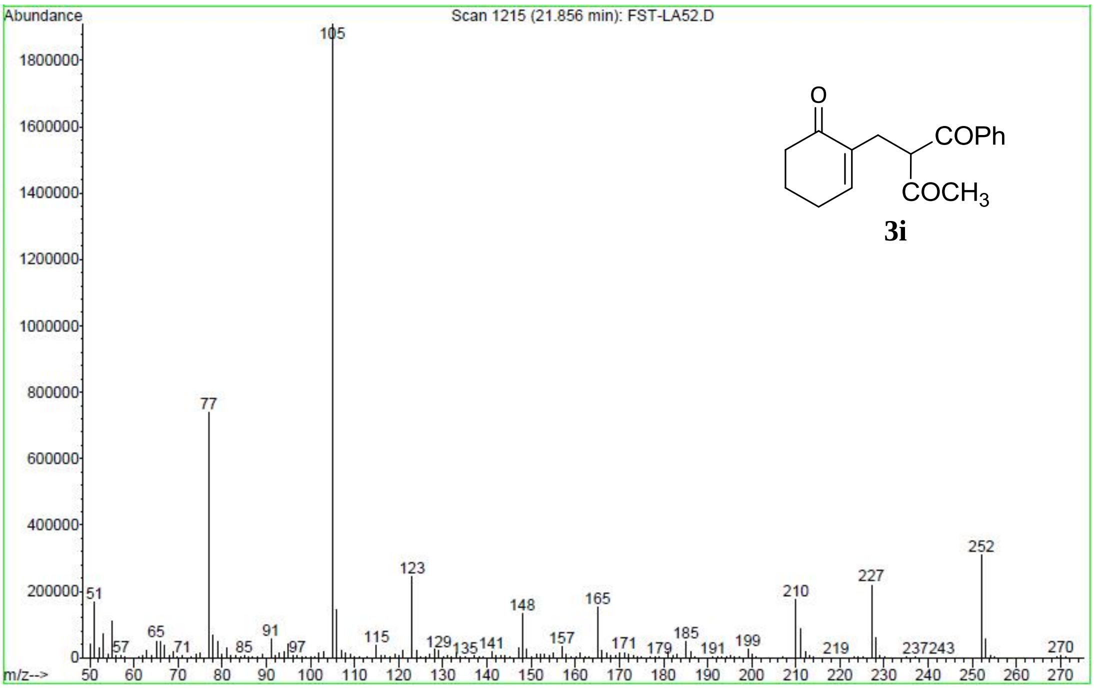
Abundance
Scan 1215 (21.856 min): FST-LA52.D
COPh
$COCH_3$
3i
105
77
123
252
227
210
165
148
51
57
65
71
85
91
97
115
129
135
141
157
171
179
185
191
199
219
237243
270
m/z-->

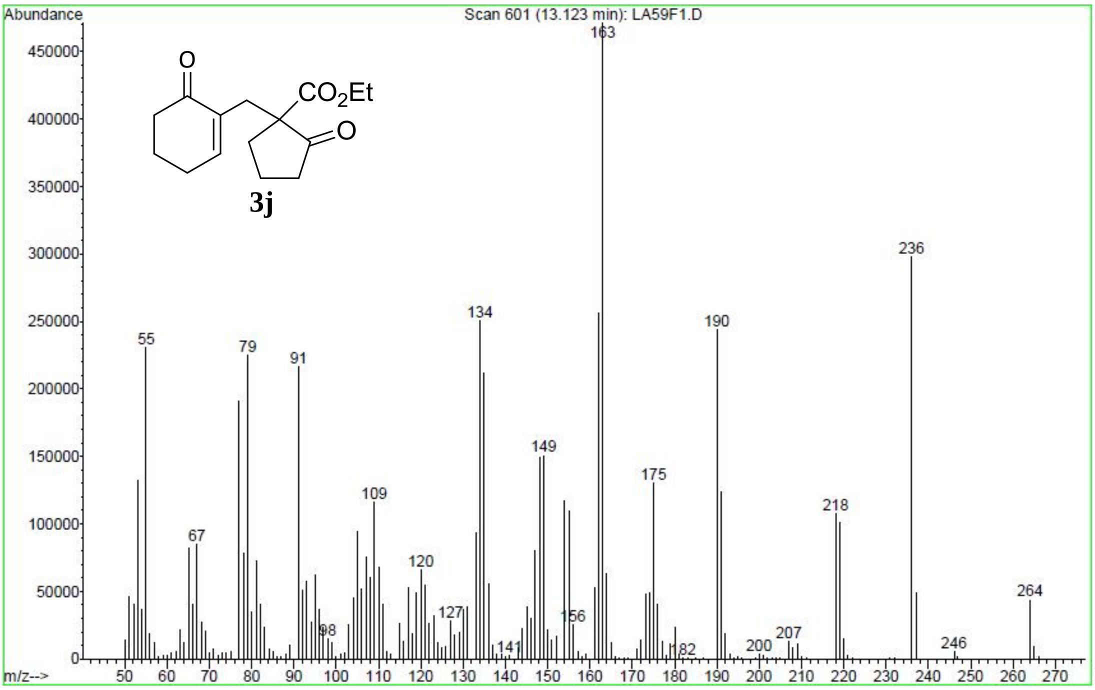
Abundance
Scan 601 (13.123 min): LA59F1.D
CO2Et
3j
55
67
79
91
98
109
120
127
134
141
149
156
163
175
182
190
200
207
218
236
246
264
m/z-->

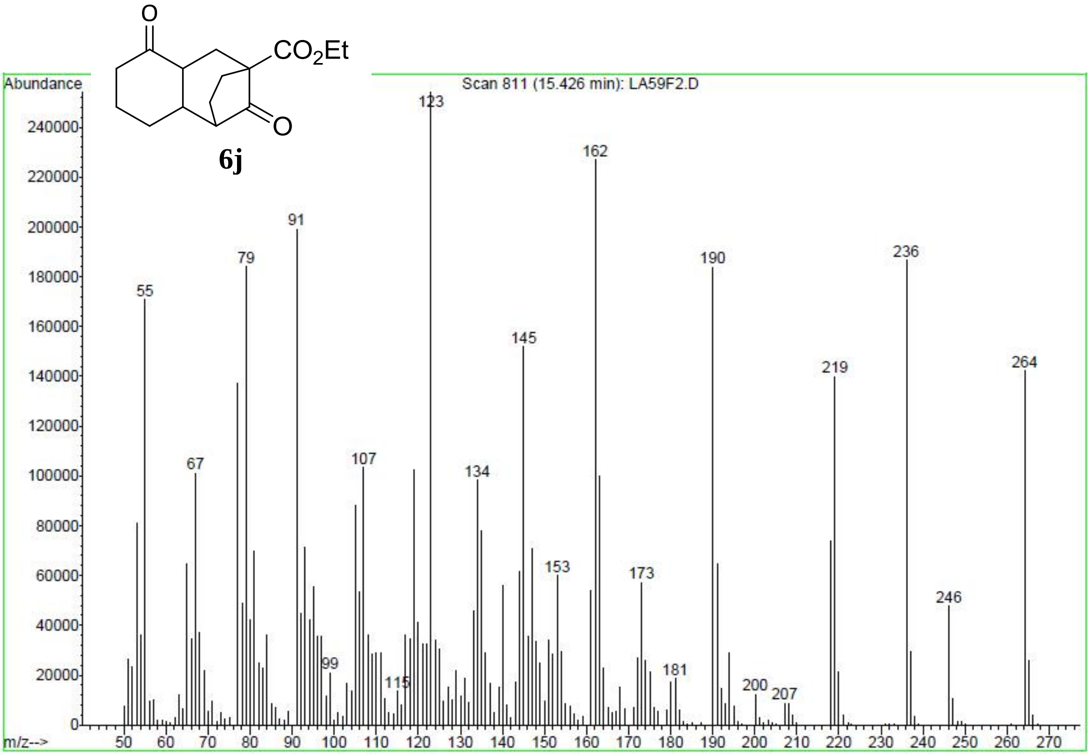
Abundance
Scan 811 (15.426 min): LA59F2.D
CO2Et
6j
55
67
79
91
99
107
115
123
134
145
153
162
173
181
190
200
207
219
236
246
264
m/z-->

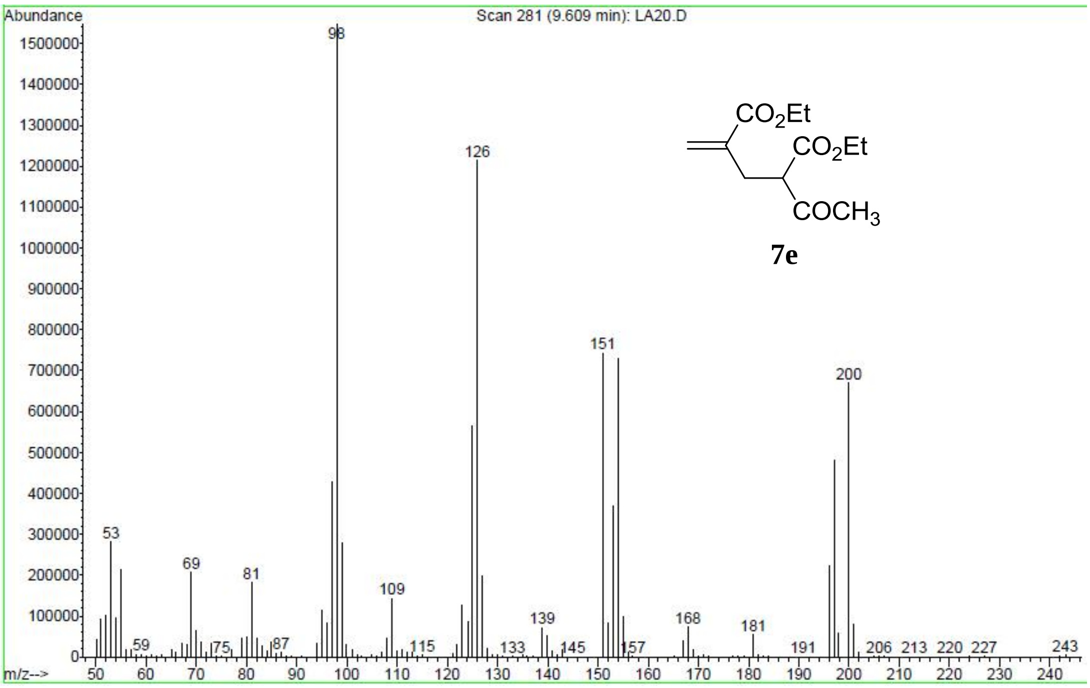
Abundance
Scan 281 (9.609 min): LA20.D
m/z-->
CO2Et
CO2Et
COCH3

7e

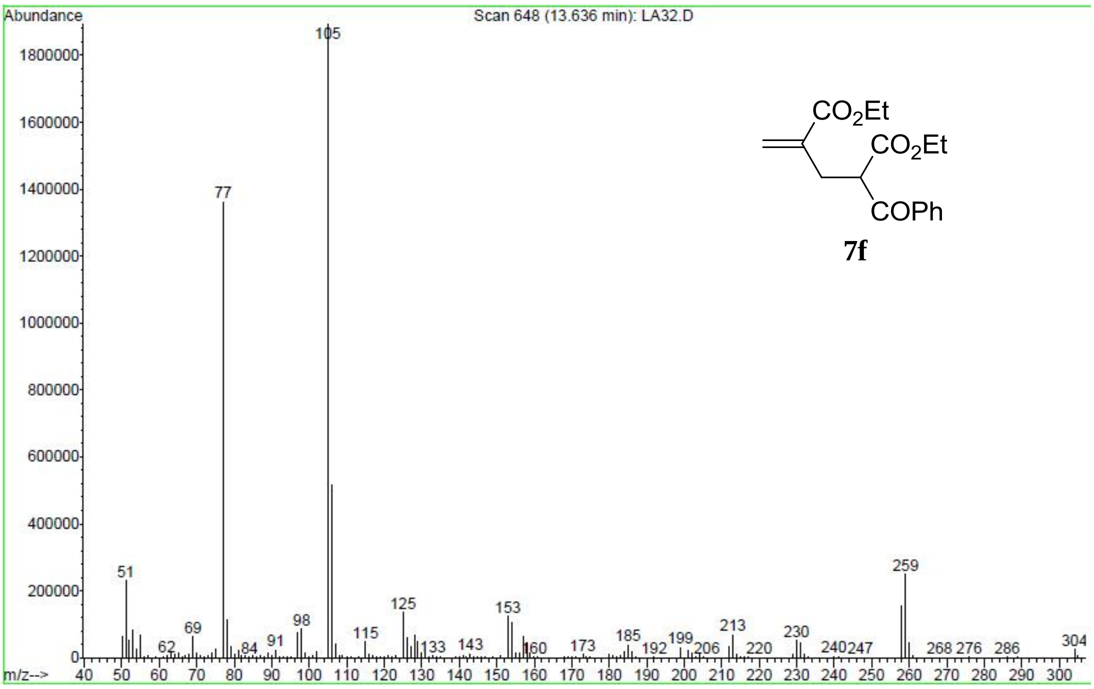
Abundance
Scan 648 (13.636 min): LA32.D
m/z-->
CO2Et
CO2Et
COPh

7f

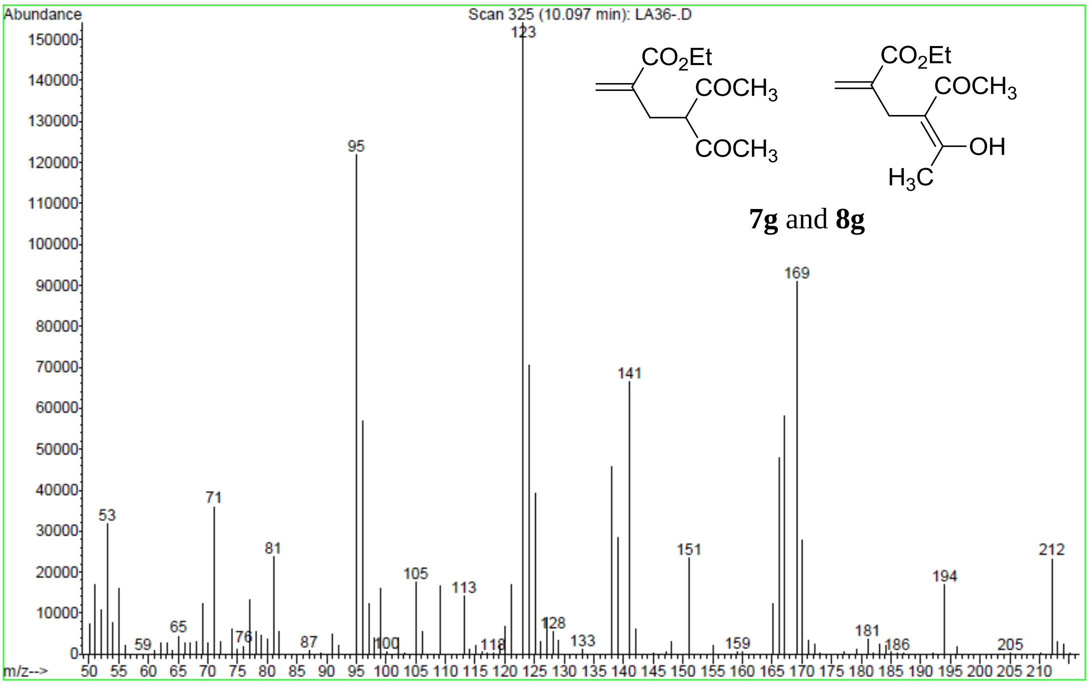
Abundance
Scan 325 (10.097 min): LA36-.D
m/z-->
CO2Et
COCH3
COCH3
CO2Et
COCH3
OH
H3C

7g and **8g**

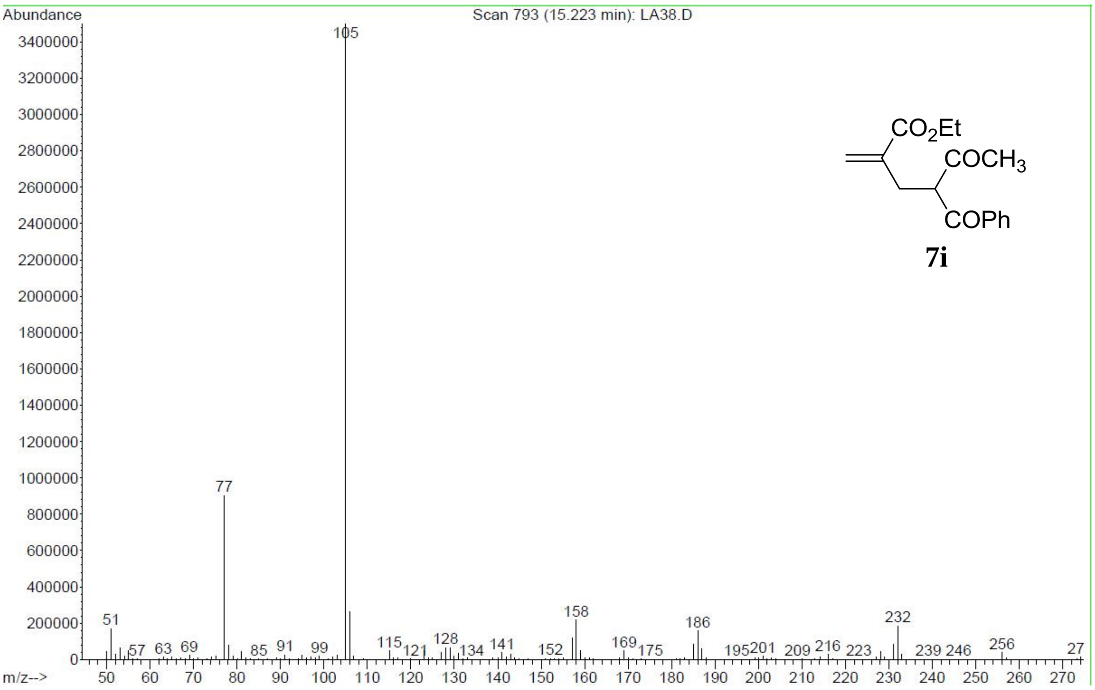
Abundance
Scan 793 (15.223 min): LA38.D
m/z-->
CO2Et
COCH3
COPh

7i

X-ray crystallographic analysis of **6j**:

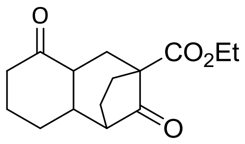

6j

supporting information

Computing details

Program(s) used to refine structure: *SHELXL97* (Sheldrick, 1997).

(ahlem)

Crystal data

$C_{15}H_{20}O_4$
$M_r = 264.31$
?, ?
$a = 9.184$ (1) Å
$b = 10.9797$ (10) Å
$c = 14.531$ (1) Å
$\alpha = 90°$
$\beta = 106.345$ (10)°
$\gamma = 90°$
$V = 1406.1$ (2) $Å^3$
$Z = 4$
$F(000) = 568$
$D_x = 1.249$ Mg m^{-3}
Mo $K\alpha$ radiation, $\lambda = 0.71073$ Å
$\mu = 0.09$ mm^{-1}
$T = 293$ K
× × mm

Data collection

Radiation source: fine-focus sealed tube
Graphite monochromator
3237 measured reflections
2903 independent reflections
1805 reflections with $I > 2\sigma(I)$
$R_{int} = 0.038$
$\theta_{max} = 27.0°$, $\theta_{min} = 2.3°$
$h = -11 \rightarrow 0$
$k = -1 \rightarrow 14$
$l = -17 \rightarrow 18$

Refinement

Refinement on F^2
Least-squares matrix: full
$R[F^2 > 2\sigma(F^2)] = 0.064$
$wR(F^2) = 0.200$
$S = 1.02$
2903 reflections
225 parameters
0 restraints
Primary atom site location: structure-invariant direct methods
Secondary atom site location: difference Fourier map
Hydrogen site location: inferred from neighbouring sites
H atoms treated by a mixture of independent and constrained refinement
$w = 1/[\sigma^2(F_o^2) + (0.0953P)^2 + 0.6309P]$ where $P = (F_o^2 + 2F_c^2)/3$
$(\Delta/\sigma)_{max} < 0.001$
$\Delta\rho_{max} = 0.41$ e $Å^{-3}$
$\Delta\rho_{min} = -0.32$ e $Å^{-3}$
Extinction correction: *SHELXL*, $Fc^* = kFc[1+0.001xFc^2\lambda^3/\sin(2\theta)]^{-1/4}$
Extinction coefficient: 0.002 (3)

References

1. Tamura, R.; Katayamaa, H.; Watabea, K.; Suzuki, H. *Tetrahedron* **1990**, *46*, 7557.
2. Rezgui, F.; El Gaïed, M. M. *Tetrahedron* **1997**, *53*, 15711.
3. Mhasni, O.; Rezgui, F. *Tetrahedron Lett*. **2010**, *51*, 586.
4. Singh, V.; Batra, S. *Synthesis* **2006**, 63.
